# Evaluation of the association between maternal folic acid supplementation and the risk of congenital heart disease: a systematic review and meta-analysis

**DOI:** 10.1186/s12937-022-00772-2

**Published:** 2022-03-26

**Authors:** Zhengpei Cheng, Rui Gu, Zenglin Lian, Harvest F. Gu

**Affiliations:** 1 School of Basic Medicine and Clinical Pharmacy, China Pharmaceutical University, Nanjing, 210009 China; 2 Strathclyde Institute of Pharmacology and Biomedical Science, University of Strathclyde, 16 Richmond St Glasgow, Glasgow, G1 1XQ Scotland; 3 College of Biotechnology and Pharmaceutical Engineering, Nanjing Tech University, Nanjing, China; 4 Institute of Biological Chinese Medicine, Beijing Yichuang Institute of Biotechnology Industry, Beijing, China

**Keywords:** Association, Atrial septal defect, Congenital heart disease, Folic acid, Heterogeneity

## Abstract

**Background:**

Folic acid (FA), as a synthetic form of folate, has been widely used for dietary supplementation in pregnant women. The preventive effect of FA supplementation on the occurrence and recurrence of fetal neural tube defects (NTD) has been confirmed. Incidence of congenital heart diseases (CHD), however, has been parallelly increasing worldwide. The present study aimed to evaluate whether FA supplementation is associated with a decreased risk of CHD.

**Methods:**

We searched the literature using PubMed, Web of Science and Google Scholar, for the peer-reviewed studies which reported CHD and FA and followed with a meta-analysis. The study-specific relative risks were used as summary statistics for the association between maternal FA supplementation and CHD risk. Cochran's *Q* and *I*^2^ statistics were used to test for the heterogeneity.

**Results:**

Maternal FA supplementation was found to be associated with a decreased risk of CHD (OR = 0.82, 95% CI: 0.72–0.94). However, the heterogeneity of the association was high (*P* < 0.001, *I*^*2*^ = 92.7%). FA supplementation within 1 month before and after pregnancy correlated positively with CHD (OR 1.10, 95%CI 0.99–1.23), and high-dose FA intake is positively associated with atrial septal defect (OR 1.23, 95%CI 0.64–2.34). Pregnant women with irrational FA use may be at increased risk for CHD.

**Conclusions:**

Data from the present study indicate that the heterogeneity of the association between maternal FA supplementation and CHD is high and suggest that the real relationship between maternal FA supplementation and CHD may need to be further investigated with well-designed clinical studies and biological experiments.

**Supplementary Information:**

The online version contains supplementary material available at 10.1186/s12937-022-00772-2.

## Introduction

Folic acid (FA) is a synthetic (that is, not generally occurring naturally) form of folate. Clinical and epidemiologic studies have demonstrated that folate deficiency during pregnancy can lead to birth defects, such as fetal neural tube defects (NTD) [1–3]. To prevent folate deficiency such as NTD, FA has been used as a substitute for natural folate because the folic acid, which due to its synthetic form has fully oxidized structure make it more stable than reduced folate [4, 5]. The preventive effect of FA supplementation in pregnant women on the occurrence and recurrence of neural tube defects (NTD) has been fully confirmed, it is generally believed that FA supplementation in pregnant women is beneficial to reproductive outcomes, including the incidence of congenital heart disease (CHD). In recent decades, however, the prevalence of CHD continues to be increased worldwide [6]. In many countries and regions in the world, the updated birth rate of CHD has become the first human birth defects, which accounts for nearly one-third of all major congenital anomalies [6, 7].

In China, the public health policy of FA supplementation for pregnant women originated from the US-China joint research project, which successfully reduced the incidence of NTD by 41–79% [8]. From 1987 to 2017, the incidence of NTD dropped from the first place among the 23 birth defects monitored during the perinatal period in China to the 12th place [9, 10]. However, with the advancement of the FA supplement policy, the overall prevalence of birth defects has not been controlled as expected, rising from 109.79/10,000 in 2000 to 153.23/10,000 in 2011 [10]. The main factor for the above deviation is increased CHD because CHD has become the largest class of birth defects since 2005 [11]. Further analysis of the birth rate of specific CHD subtypes showed that the incidence of atrial septal defect (ASD) has increased significantly over time [12]. We are unable to explain the differences in temporal trends in incidence between NTD and CHD. Apparently, there is a large discrepancy between the current state of FA supplementation to prevent CHD knowledge and practice for clinical application. Therefore, the question of whether there exists an association between maternal FA supplementation and the risk of CHD is raised. To address this question, in the present study, we have conducted a systematic review and meta-analysis to evaluate the association of maternal FA supplementation on the risk of CHD, and to provide a scientific basis for further medical decision and research on maternal FA supplementation and prevention of CHD.

## Methods

### Search strategy

We searched the literature with a cut-off date of June 30, 2021, using PubMed, Web of Science and Google Scholar, for the peer-reviewed studies with English abstracts which reported CHD and FA. The main search terms used were (‘congenital heart disease’, or ‘congenital heart defect’, or ‘CHD’, or ‘septal defect’ or ‘atrial septal defect’) and (‘folic acid’, or ‘folate’, or ‘multivitamins’). In addition, we searched for the studies that using the key words of coronary artery disease and birth defects and examined the relevant references. We further followed published quality standards for conducting the meta-analyses [13].

### Eligibility criteria

We selected the articles that (1) were original epidemiologic studies and clinical control studies (i.e., case–control, cohort or randomized controlled trial, RCT), (2) examined the association between periconceptional FA use and either CHD overall or ASD (or septal defects) subtypes in infants, (3) were published in the English language, (4) either the results reported as risk ratios or odds ratios (OR) and 95% confidence intervals (CI) or provided raw data from which these measures could be calculated, (5) defined CHD or ASD (or septal defects) subtypes as an outcome. We also excluded the results of research in the pregnant women with diseases (for instance, diabetes). In addition, non-peer reviewed articles and the studies with experimental animals, concerning ecological assessments, and mechanisms were excluded. Articles that reported results contained multiple populations were considered to consist of separate studies, with one study for each population investigated. Only first published article or the largest number of cases was included in our study, when multiple articles were found to examine the same study.

### Date extraction

Data extraction and quality assessment were completed independently by two researchers to reduce the bias and errors of in the date extraction process. Disagreements between investigators were resolved through discussions until a consensus was reached. The following study characteristics were recorded: publication year, geographic region, the sample size, case classification information, exposure, and outcome assessments, adjusted estimates and their corresponding 95% Cl and confounding factors that were controlled for by adjustments in the data analysis. Because a proportion of women might take a multivitamin containing FA during pregnancy, in the current study, we thus analyzed the data separately and found similar results when taking FA alone or taking a multivitamin (Please see Supplemental Fig. 1). If the content of FA in the multivitamin could not be determined to be 0.4 mg, we did not include these data in the analysis. Additionally, the information on the dosage and the timing of FA intake were collected, grouped into three types of FA dose: high-dose, medium-dose, and low-dose. We assessed initiation of any folic intake for 3 times window: 4 weeks before conception, 4 weeks after conception, 5 to 12 weeks after conception. Some studies did not report the exact time of initiation of FA intake, we divided it into four types: short-term taking before pregnancy, long-term taking before pregnancy, short-term taking after pregnancy, long-term taking after pregnancy. To assess study quality, we used a 9-star system based on the Newcastle–Ottawa Scale [14]. We defined a high-quality study as one with a quality score greater than or equal to 7.

**Fig. 1:**
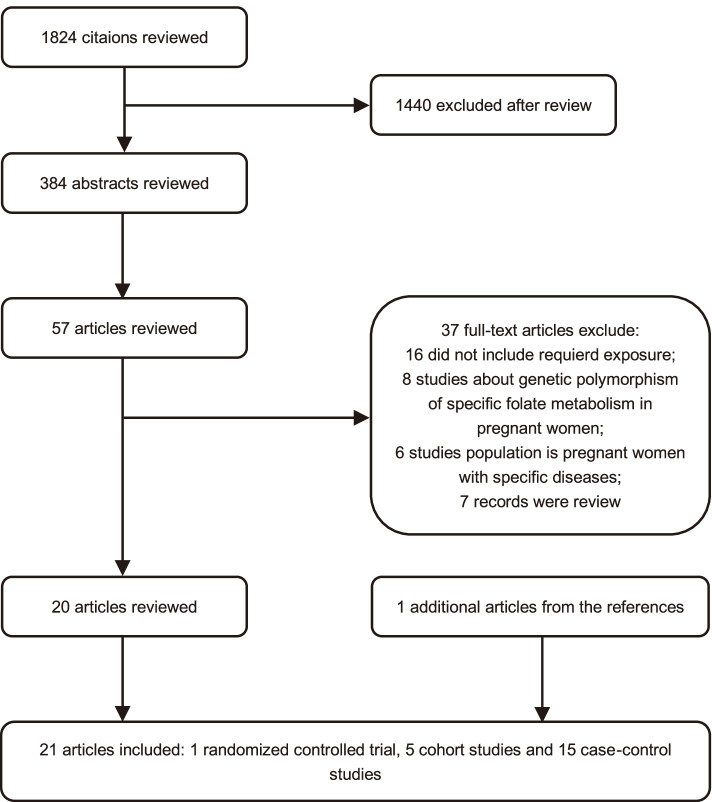
A flow chart of identification and selection of the studies in the current meta-analysis

### Statistical analysis

As summary statistics, we used study-specific relative risks for the association between maternal FA supplementation and CHD risk. To simplify the procedure, an OR was used to represent results from case–control studies and an RR was used to represent all reported study-specific results from cohort studies. Heterogeneity among studies was evaluated using Cochran's *Q* and *I*^2^ statistics.

We conducted subgroup analyses based on study design (i.e., RCT or cohort versus case–control studies), geographical region (i.e., USA, Europe, and China), study quality (i.e., low versus high quality) and relevant confounder (i.e., age). In addition, we have made statistics on the time and dose of FA intake. We evaluated heterogeneity between subgroups by meta-regression analysis. A *P*-value less than 0.05 for the meta-regression analysis was considered to indicate a significant difference between subgroups.

Publication bias was assessed by via visual inspection of a funnel plot for asymmetry using Egger's linear regression [15] and Begg's rank correlation methods [16]. For both tests, significant statistical publication bias was defined to be indicated by a *P*-value of < 0.05. All statistical analyses were performed using STATA software (version 14.0; StataCorp, College Station, Texas, USA).

## Results

### Study characteristics

The initial search returned a total of 1,824 potentially eligible publications from databases. Finally, a total of 21 studies involving 106,920 CHD individuals were included for the analysis. The process of identification and selection in the studies for the current meta-analysis is summarized in Fig. 1. All the studies were published in the period from 1993 to 2020, and they included 1 randomized controlled trial, 5 cohort studies and 15 case–control studies. The characteristics of all studies are summarized in Supplemental Table 1. Of them, more than half of the studies (61.9%) were published after 2010, and 5 studies performed in the United States [17–21], 9 studies in Europe [22–30], 4 studies in China [31–34], 2 studies in Canada [35, 36], and 1 study in Australia [37].

**Table 1:** Dosage information in FA supplementation and it’s relation with CHD or ASD

Authors (year)	Intake of folic acid	CHD OR (95% Cl)	ASD OR (95% Cl)
Scanlon KS et al. (1998) [18]	Low-dose group < 245 μg	1.11 (0.63, 2.00)	
	Middle-dose group 245–355 μg	1	
	High-dose group 356–542 μg	1.30 (0.78, 2.22)	
Shaw GM et al. (2010) [20]	Low-dose group < 293.8 μg	1.45 (0.87, 2.41)	
	Middle-dose group 293.8–546.3 μg	1	
	High-dose group ≥ 546.4 μg	0.74 (0.41, 1.31)	
Czeizel AE et al. (2015) [25]	Low-dose group 3 mg	1.06 (0.78, 1.44)	1.49 (0.65, 3.41)
	Middle-dose group 6 mg	1	1
	High-dose group 9 mg	1.14 (0.71, 1.81)	1.81 (0.51, 6.42)
Mao B et al. (2017) [34]	Low -dose group < 149.88 μg	1.63 (1.01, 2.62)	1.44 (0.72, 2.87)
	Middle-dose group 149.88–266.35 μg	1	1
	High-dose group ≥ 266.35 μg	1.06 (0.62, 1.81)	1.07 (0.50, 2.25)

*FA* folic acid, *CHD* congenital heart disease, *ASD* atrial septal defects

### Maternal FA supplementation and CHD

**Fig. 2:**
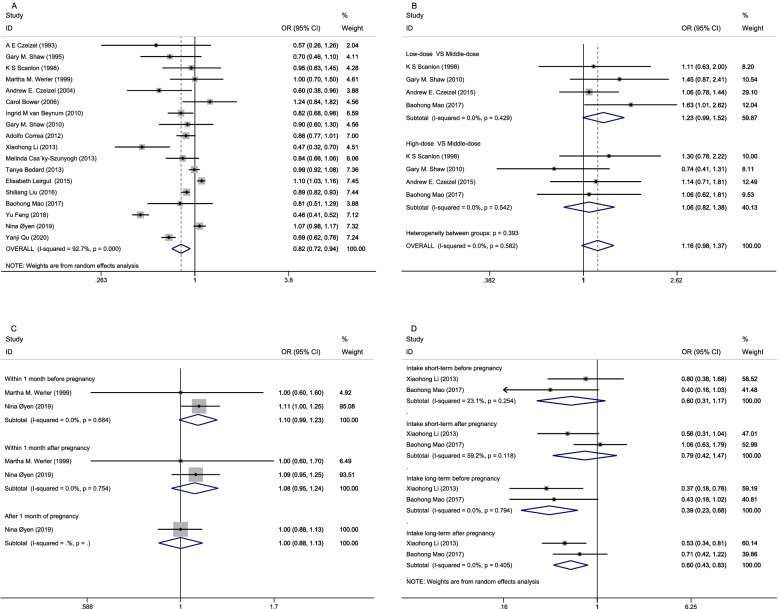
Possible association between maternal FA supplementation and CHD. FA: folic acid; CHD: congenital heart disease; A. Odd ratio (OR) estimates for the overall association of maternal FA supplementation with the risk of CHD; B. OR estimates for the association between maternal FA supplement intake and CHD; C. OR estimates for the association between the initiation of FA supplementation on CHDs; D. Estimated OR of the association between FA supplementation time and CHD

Figure 2 represents the results of the association between maternal FA supplementation and the risk of CHD in this study. The overall results of this meta-analysis showed the decreased risk of CHD with maternal FA supplementation (OR = 0.82, 95% CI: 0.72–0.94; Fig. 2A). Figure 2B shows the comparison results with each dose level of the maternal FA supplementation and the association of CHD. Of the 21 studies included in the current study, data from 4 studies were adopted to evaluate CHD for the intake of FA during pregnancy. Each of the studies included in FA intake is not consistent, we thus summarized and roughly divided into high-dose group, low-dose group, and middle-dose group, while the middle-dose group was taken into OR calculation as a reference. FA dosage information included in these studies is summarized and represented in Table 1. The results of this meta-analysis provided evidence that FA intake affected the association of CHD. The summary OR for any type of heart defect of low dose intake compared with middle dose intake was 1.23 (95% CI, 0.99–1.52), with no significant heterogeneity between studies (*I*^2^ = 0%, *P* = 0.429); the high-dose group was 1.06 (95% CI, 0.82–1.38), with no significant heterogeneity between studies (*I*^2^ = 0%, *P* = 0.542).

As presented in Fig. 2C, the meta-analysis showed that the initiation of FA supplementation within 1 month before conception and 1 month after conception was associated with an increased risk of CHD, the ORs of any heart defect in offspring was 1.10 (95% CI, 0.99–1.23), 1.08 (95% CI, 0.95–1.24), compared with the reference group with no FA intake. Only 4 studies assessed the effects of time of pregnant women FA exposure on CHD [19, 22, 31, 34]. With above studies, 2 studies evaluated initiation of any FA intake within one month before or after pregnancy [19, 22], one month after pregnancy, the remained 2 studies evaluated the effect of maternal FA supplementation on CHD by the length of time [31, 34]. The association between the duration of maternal FA supplementation and CHD occurrence is summarized in Table 2. Data from the meta-analysis demonstrated that the association between short-term FA supplementation after pregnancy was weakest. The OR of any heart defect in offspring was 0.79 (95% CI, 0.42–1.47) compared with the reference group with no FA intake (Fig. 2D).

**Table 2:** Association between duration of maternal FA supplementation and occurrence of CHD or ASD

Folic acid intake	Authors (year)	Duration	CHD OR (95% Cl)	ASD OR (95% Cl)
Intake short-term before pregnancy	Li X et al. (2013) [31]	Before pregnancy ≤ 1 month	0.60 (0.31, 1.17)	0.53 (0.27, 1.05)
	Mao B et al. (2017) [34]	Before pregnancy ≤ 2 months		
Intake short-term after pregnancy	Li X et al. (2013) [31]	During pregnancy < 1 month	0.79 (0.42, 1.47)	0.66 (0.41, 1.07)
	Mao B et al. (2017) [34]	During pregnancy ≤ 3 months		
Intake long-term before pregnancy	Li X et al. (2013) [31]	Before pregnancy 1–3 months	0.39 (0.23, 0.68)	0.28 (0.13, 0.58)
	Mao B et al. (2017) [34]	Before pregnancy > 2 months		
Intake long-term after pregnancy	Li X et al. (2013) [31]	During pregnancy > 1 months	0.60 (0.43, 0.83)	0.43 (0.26, 0.70)
	Mao B et al. (2017) [34]	During pregnancy ≥ 3 months		

*FA* folic acid, *CHD* congenital heart disease, *ASD* atrial septal defects

### Maternal FA supplementation and ASD

**Fig. 3:**
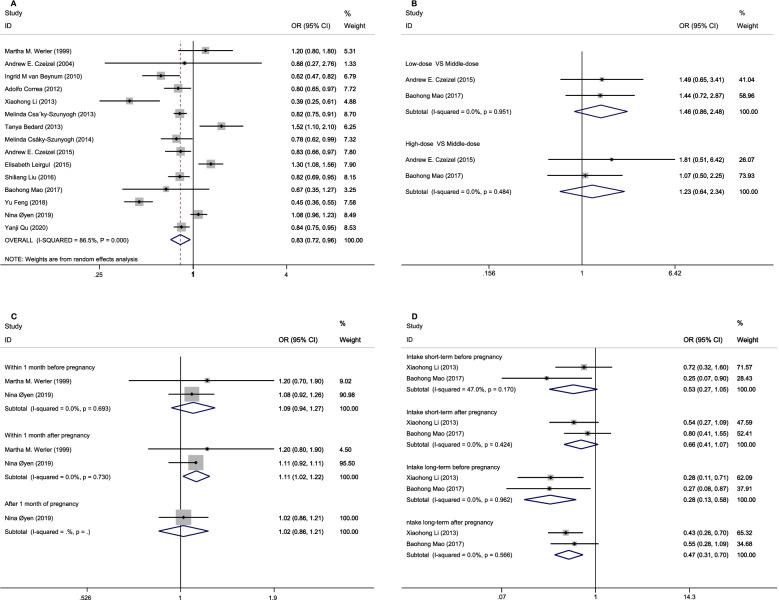
Possible association between maternal FA supplementation and ASD. FA: folic acid; ASD: atrial septal defects; A. Odd ratio (OR) estimates for the overall association of maternal FA supplementation with the risk of ASD; B. OR estimates for the association between Maternal folic acid supplement intake and ASD; C. OR estimates for the association between the initiation of folic acid supplementation on ASD; D. Estimated OR of the association between folic acid supplementation time and ASD

As seen in Fig. 3, there was the association between maternal FA supplementation and the risk of ASD in this study. The overall results of this meta-analysis showed a decreased in the risk of ASD (or septal defect) with maternal FA supplementation (OR 0.83, 95% CI: 0.72–0.96; Fig. 3A). The summary OR for ASD (or septal defect) of low dose intake compared with middle dose intake was 1.46 (95% CI, 0.86–2.48); the high-dose group was 1.23 (95% CI, 0.64–2.34), with no heterogeneity (Fig. 3B). FA dosage information included in these studies is summarized and represented in Table 1.

The initiation of FA supplementation was represented by three variables: within 1 month before pregnancy, within 1 month after pregnancy, after 1 month of pregnancy, the ORs of ASD in offspring was 1.09 (95% CI, 0.94–1.27), 1.11 (95% CI, 1.02–1.22), 1.02 (95% CI, 0.86–1.21) compared with the reference group with no FA intake (Fig. 3C). The initiation of FA supplementation within 1 month after conception was associated with an increased OR for ASD. Data from the meta-analysis demonstrated that the association between short-term FA supplementation after pregnancy was weakest. The OR of ASD in offspring was 0.66 (95% CI, 0.41–1.07) compared with the reference group with no FA intake (Fig. 3D). The association between the duration of maternal FA supplementation and CHD occurrence is summarized in Table 2.

### Heterogeneity analysis

**Fig. 4:**
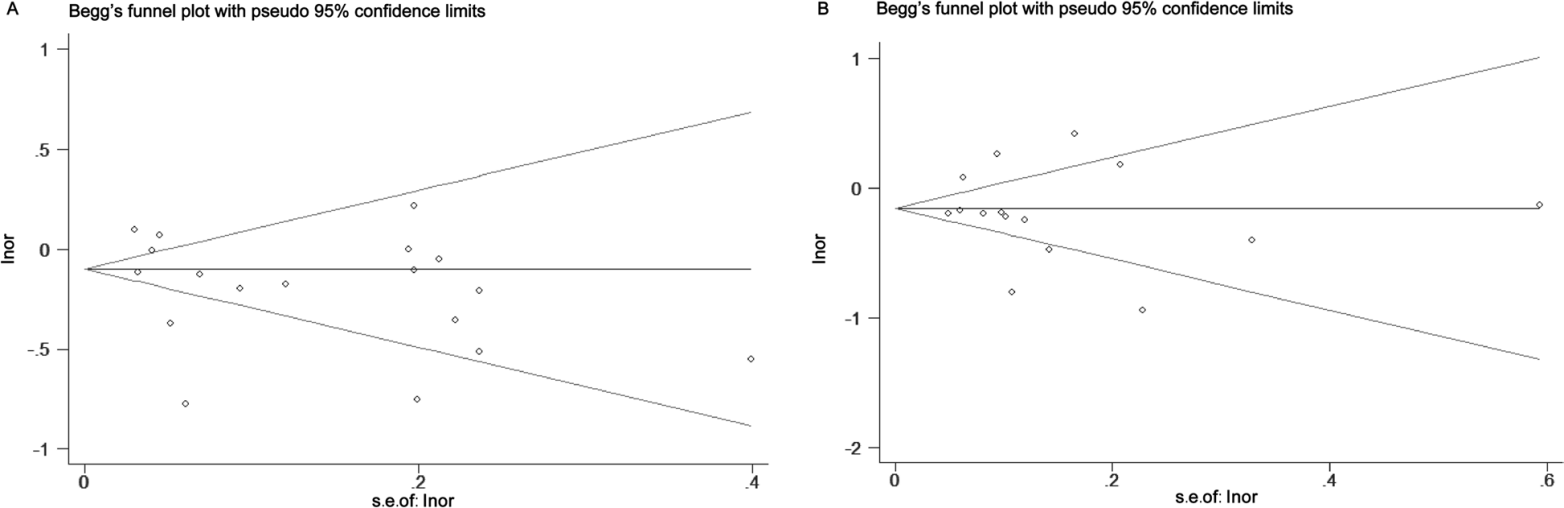
Begg’s test for possible association between FA supplementation and CHD/ASD. FA: folic acid; CHD: congenital heart disease; ASD: atrial septal defects; A. Begg's test of studies examining the association between maternal folate supplementation and the risk of CHD; B. Begg's test of studies examining the association between maternal folate supplementation and the risk of ASD

Although our meta-analysis showed that maternal FA supplementation reduced the risk of CHD and ASD (or septal defect), we found that the heterogeneity of studies for possible association between FA supplementation and CHD was significant (*P* < 0.001, *I*^*2*^ = 92.7%), with no publication bias (Begg’s test: *P* = 0.211; Fig. 4A). Furthermore, a significant heterogeneity of studies for possible association between FA supplementation and ASD was also detected (*P* < 0.001, I^2^ = 86.5%), with no publication bias (Begg’s test: *P* = 0.621; Fig. 4B).

To clarify the sources of heterogeneity, we conducted a sensitivity analysis. However, *I*^*2*^ did not decrease substantially when any individual study was removed. Subsequently, a subgroup analysis was carried out on the studies of CHD and maternal FA supplementation. In the subgroup analyses, the corresponding pooled OR was not materially altered in any stratification (Please see Supplemental Figs. 2 and 3, Table 3). However, the results of the meta-analysis have changed in different regions, different designs. Studies in Europe/Australia showed that there was no statistical difference between FA supplementation and the incidence of CHD (OR 0.96, 95%CI 0.84–1.09), and the cohort study/RCT study showed that there was no statistical difference between FA supplementation and the incidence of CHD (OR 0.94, 95%CI 0.84–1.05) but there is still significant heterogeneity between studies. We also found that the differences in age, maternal smoking, family history may contribute to the heterogeneity.

**Table 3:** The subgroup analyses of studies concerning the association between maternal FA supplementation and the risk of CHD in offspring

Subgroup	No. of studies	OR (95% CI)	*P-*value	*I*^*2*^(%)
Summary pooled estimate	18	0.821 (0.720, 0.936)	< 0.001	92.7
Design				
Case–control	12	0.801 (0.648, 0.991)	< 0.001	94.7
Cohort or RCT	6	0.936 (0.837, 1.047)	0.003	72.2
Geographic region				
Europe or Australia	7	0.955 (0.840, 1.086)	0.001	72.1
China	4	0.580 (0.433, 0.776)	< 0.001	89.6
America	7	0.921 (0.874, 0.971)	0.358	9.3
Publication period				
Before 2010	5	0.927 (0.737, 0.936)	0.253	25.2
2010 or after	13	0.800 (0.690, 0.928)	< 0.001	94.7
Quality assessment				
High quality studies (scores ≥ 7)	11	0.803 (0.641, 1.007)	< 0.001	95.5
Low quality studies (scores < 7)	7	0.898 (0.832, 0.969)	0.120	40.6
Confounding Factors				
Age	14	0.821 (0.720, 0.936)	< 0.001	94.2
Maternal smoking	5	0.751 (0.562, 1.005)	< 0.001	94.9
Maternal alcohol	2	0.801 (0.677, 0.948)	0.512	0
Maternal BMI	5	0.829 (0.676, 1.016)	< 0.001	83
Fetal sex	2	0.806 (0.654, 0.992)	0.471	0
Family history	2	0.726 (0.324, 1.623)	< 0.001	93.8

*FA* folic acid, *CHD* congenital heart disease

Table 4 represents the subgroup analyses of studies examining the association between maternal FA supplementation and the risk of ASD (or septal defect) in offspring. We found that differences in the geographical region, design, age, Maternal smoking, family history may contribute to the heterogeneity we observed. In subgroup analyses, the corresponding pooled ORs for studies of “Cohort or RCT”, “America”, “Before 2010” were 1.010, 1.008, 1.200, and were materially altered (Table 4 and see Supplemental Figs. 4 and 5). Design of the studies may change the results of association between maternal FA supplementation and ASD. However, RCT is believed to yield the highest level of evidence for causality because of no recall bias and other advantages. Moreover, the prevalence of ASD was shown an upward trend since 2009 [6]. Therefore, the effect of maternal FA supplementation to prevent ASD may be still under question.

**Table 4:** The subgroup analyses of studies regarding the association between maternal FA supplementation and the risk of ASD in offspring

Subgroup	No. of studies	OR (95%CI)	*P-*value	*I*^*2*^(%)
Summary pooled estimate	15	0.828 (0.716, 0.957)	< 0.001	86.5
Design				
Case–control	10	0.768 (0.646, 0.914)	< 0.001	87.7
Cohort or RCT	5	1.010 (0.789, 1.291)	0.004	74
Geographic region				
Europe or Australia	7	0.892 (0.748, 1.065)	< 0.001	83.4
China	4	0.564 (0.362, 0.879)	< 0.001	90.8
America	4	1.008 (0.766, 1.327)	0.002	79.5
Publication period				
Before 2010	1	1.200 (0.800, 1.800)		
2010 or after	14	0.811 (0.698, 0.941)	< 0.001	87.1
Quality assessment				
High quality studies (scores ≥ 7)	10	0.789 (0.652, 0.954)	< 0.001	90.0
Low quality studies (scores < 7)	5	0.908 (0.730, 1.130)	0.009	70.2
Confounding Factors				
Age	13	0.795 (0.687, 0.919)	< 0.001	86.8
Maternal smoking	4	0.748 (0.512, 1.094)	< 0.001	91.8
Maternal alcohol	1	0.620 (0.469, 0.819)		
Maternal BMI	5	0.704 (0.508, 0.975)	< 0.001	87
Fetal sex	1	0.780 (0.617, 0.986)		
Family history	2	0.664 (0.245, 1.801)	< 0.001	94.6

*FA* folic acid, *ASD* atrial septal defects

## Discussion

We have conducted a meta-analysis of the recent 21 studies concerning maternal FA supplementation and CHD. Although the data from our analysis implicate that maternal FA supplementation is associated with the reduced risk of CHD, the heterogeneity of this association is high. First, the association is presented to be geographically different. The reports from China suggest that FA supplementation is associated with the decreased incidence of CHD [31–34], while the studies in Europe and Australia demonstrate that the association was not statistically significant [22–30, 37]. Second, the designs of these studies are related with the different results. The case–control studies implicate that FA supplementation reduced the incidence of CHD, but the cohort/RCT studies indicate no significant association [22, 29, 30, 34–36]. There could be several explanations for the high heterogeneity. First, the baseline FA levels among fertile women in the developing regions or countries are lower. Second, the case–control studies are observational and may not provide the evidence as cohort study/RCT at the same levels [38] because the case–control study may be confounded by the factors, including age, maternal smoking, maternal alcohol, maternal BMI fetal sex, and family history. Indeed, confounding is always an issue when assessing the association of a single environmental factor with a complex outcome like CHD, particularly, the incidence of CHD is low [6]. Third, the dosage of FA could be one more issue for these conflicting results.

According to clinical phenotypes, ASD is classified as one of subtypes of CHD [39]. In the current study, we found that the summary OR of ASD at the high-dose group of FA intake was increased by nearly 20% compared with what at the middle dose group. It is interesting that we found a positive association between FA supplementation within 1 month before and after pregnancy and CHD, and high-dose FA intake is positively associated with ASD. FA supplementation may have a negative effect on heart development in high doses or specific time windows, the precise biological mechanisms of FA on heart development remain to be elucidated [40]. At present, little is known about the effects of dosage of FA supplementation on infant birth outcome. However, several studies have reported the association between the dosage of FA supplements and adverse pregnancy outcomes. A cohort reported that FA supplement use ≥ 800 µg/day during pregnancy was related to elevated gestational diabetes mellitus risk [41]. In addition, the use of high-dose (≥ 800 µg/d) FA supplements is associated with an increased risk of gestational hypertension [42], FA also affect fetal cardiovascular system [43].

In epidemiological studies, there are many factors that may affect the analysis of the results and the results of the analysis. Differences in methods used for studies are probably the most common factor contributing to heterogeneity. Some scholars have proposed that the addition of multivitamins containing folic acid may improve the effect of primary prevention of CHD compared with the use of FA alone [44]. To exclude interfering factors, in the current study, we not only analyzed taking FA alone and taking multivitamins containing FA, but also excluded the content of FA in multivitamins below 0.4 mg from the analysis. By using zebrafish, an experimental study has demonstrated that maternal micronutrient and homocysteine status are associated with the risk of CHD in offspring [45]. Furthermore, FA and other micronutrient deficiencies can lead to accumulation of homocysteine and mitotic dysfunction [45, 46]. In China, after the implementation of public health policies to increase FA intake in women, population statistics should theoretically support the preventive effect of FA intake during pregnancy on CHD, but the results are not consistent with expectations [11]. This problem cannot be fully explained, as more than 90% of women take supplements [47]. Therefore, through the analysis progress of this study, we believe that due to the different methods of taking FA to avoid the generation of heterogeneity, it may be more important to strengthen the within-group comparison than the between-group comparison in the analysis method and process.

Several studies have demonstrated that the continued FA supplementation after pregnancy may increase the risk of large-for-gestational-age birth [48], childhood asthma [49], childhood allergic [50], negative neurodevelopmental outcomes [51, 52]. Thereby, we have an assumption that FA supplementation may not negatively but positively be associated with the risk of CHDs was consistent with the trend of CHD epidemic. Further research should be done on the reasonable time and dosage of FA intake and the mechanism of adverse pregnancy caused by excessive supplementation.

## Conclusion

Data from the current meta-analysis suggest that although the maternal FA supplementation seems associated with a decreased risk of CHD, the heterogeneity of this association is significantly high. The heterogeneity may be caused by the confounders such as timing and dose of FA administration, and the heterogeneity may subsequently influence the outcome on actual effect of FA supplementation on CHD. On this basis, we believe it is necessary to correctly assess the association of FA supplementation with CHD. Further experiments designed to study the association between FA and CHD and its molecular mechanisms have been taken into consideration.

## Supplementary Information

**Additional file 1.**

**Additional file 2.**

**Additional file 3.**

**Additional file 4.**

**Additional file 5.**

## Data Availability

All the data in this study were available in the figures in the main text and supplemental documents of this manuscript.

## Abbreviations

ASD: Atrial septal defect
BMI: Body mass index
CHD: Congenital heart disease
FA: Folic acid
NTD: Neural tube defect
OR: Odds ratio
RR: Risk ratio
THF: Tetrahydrofolate
VSD: Ventricular septal defect
