# Supplementary figures and images for: Evaluation of the association between maternal folic acid supplementation and the risk of congenital heart disease: a systematic review and meta-analysis
Source: Nutr J. 2022 Mar 26;21:20. doi: 10.1186/s12937-022-00772-2 (PMC8962131; doi:10.1186/s12937-022-00772-2)

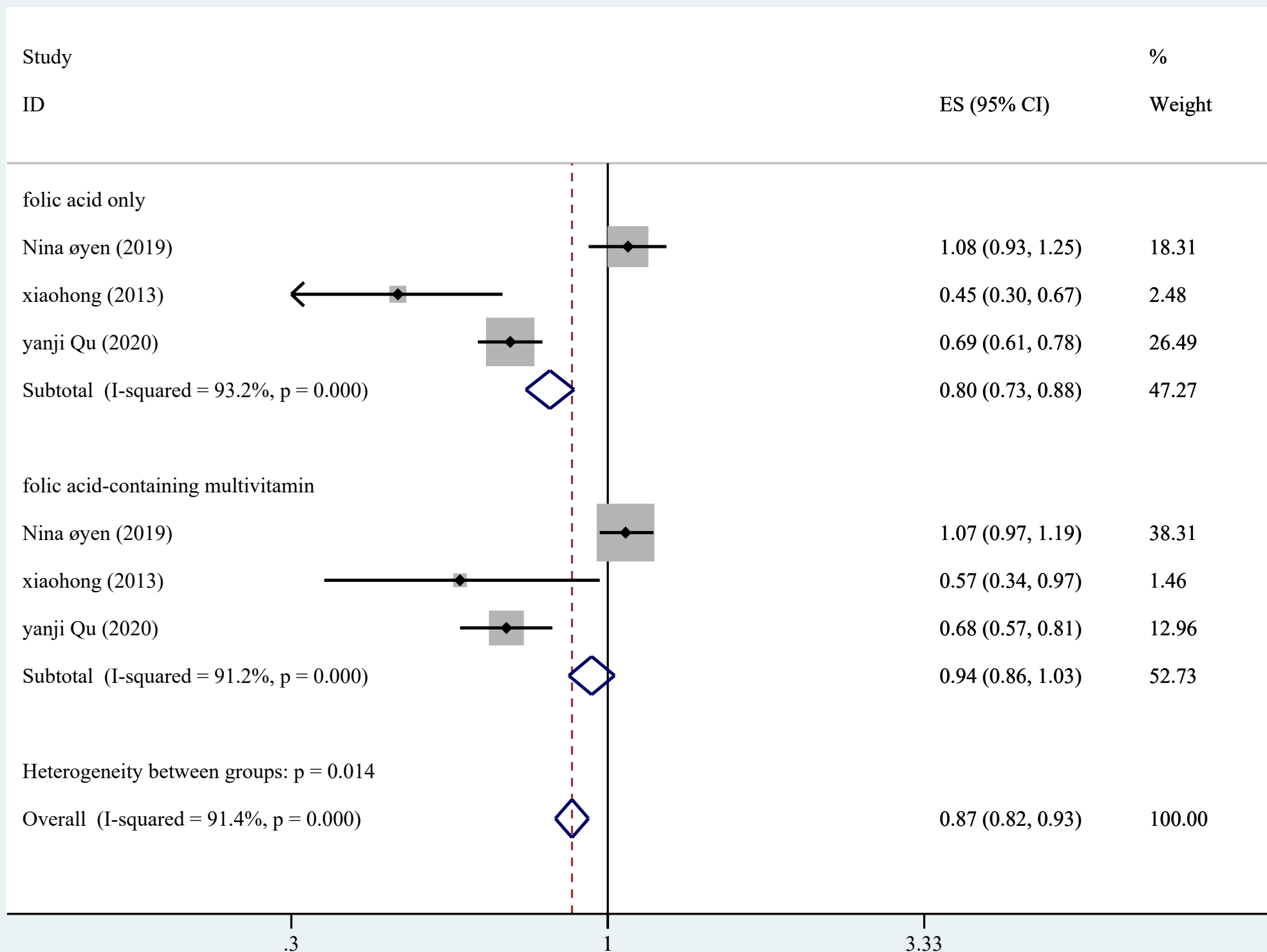

Supplement: Supplementary file 1 — Additional file 1. [file 12937_2022_772_MOESM1_ESM.pdf]

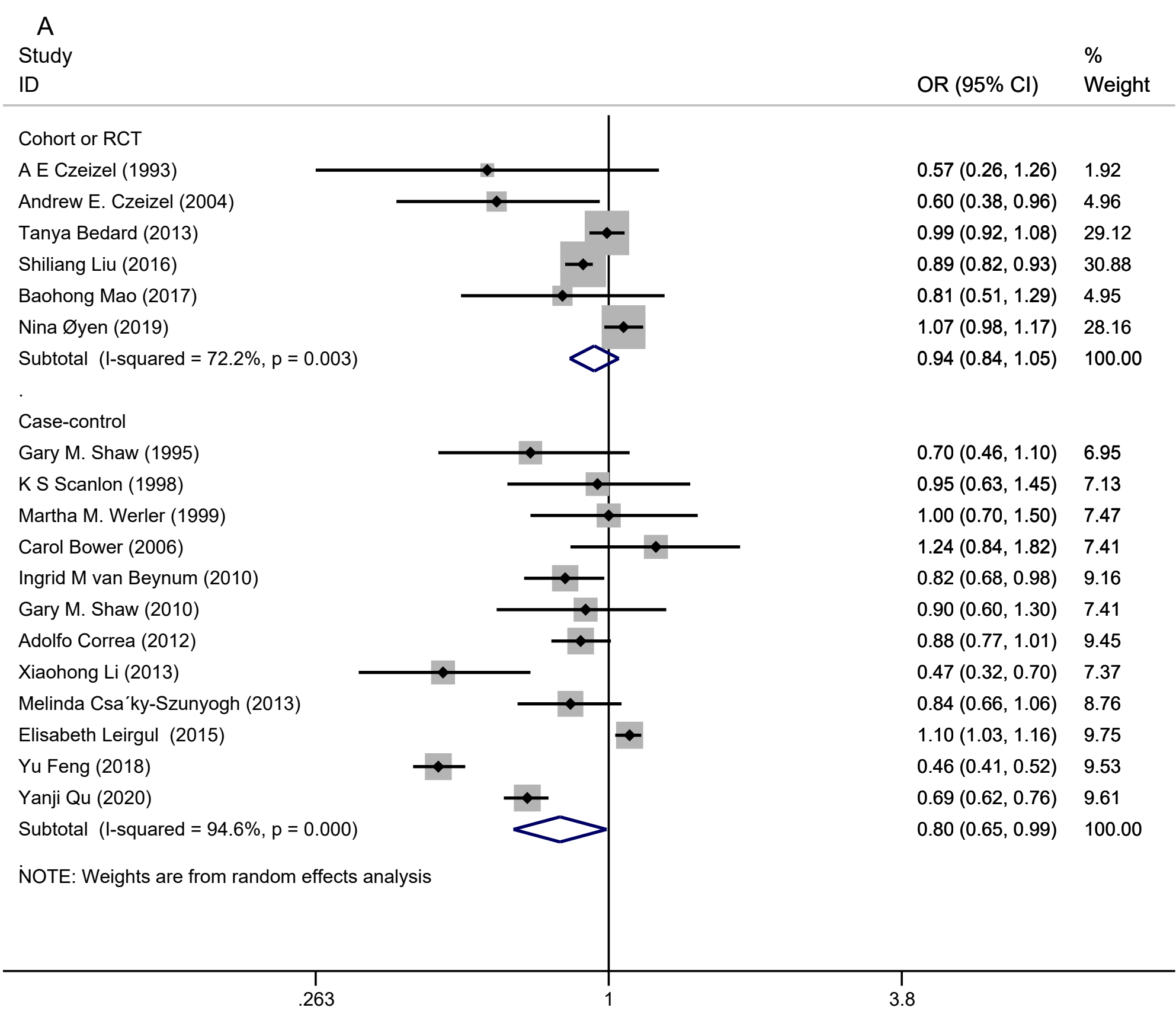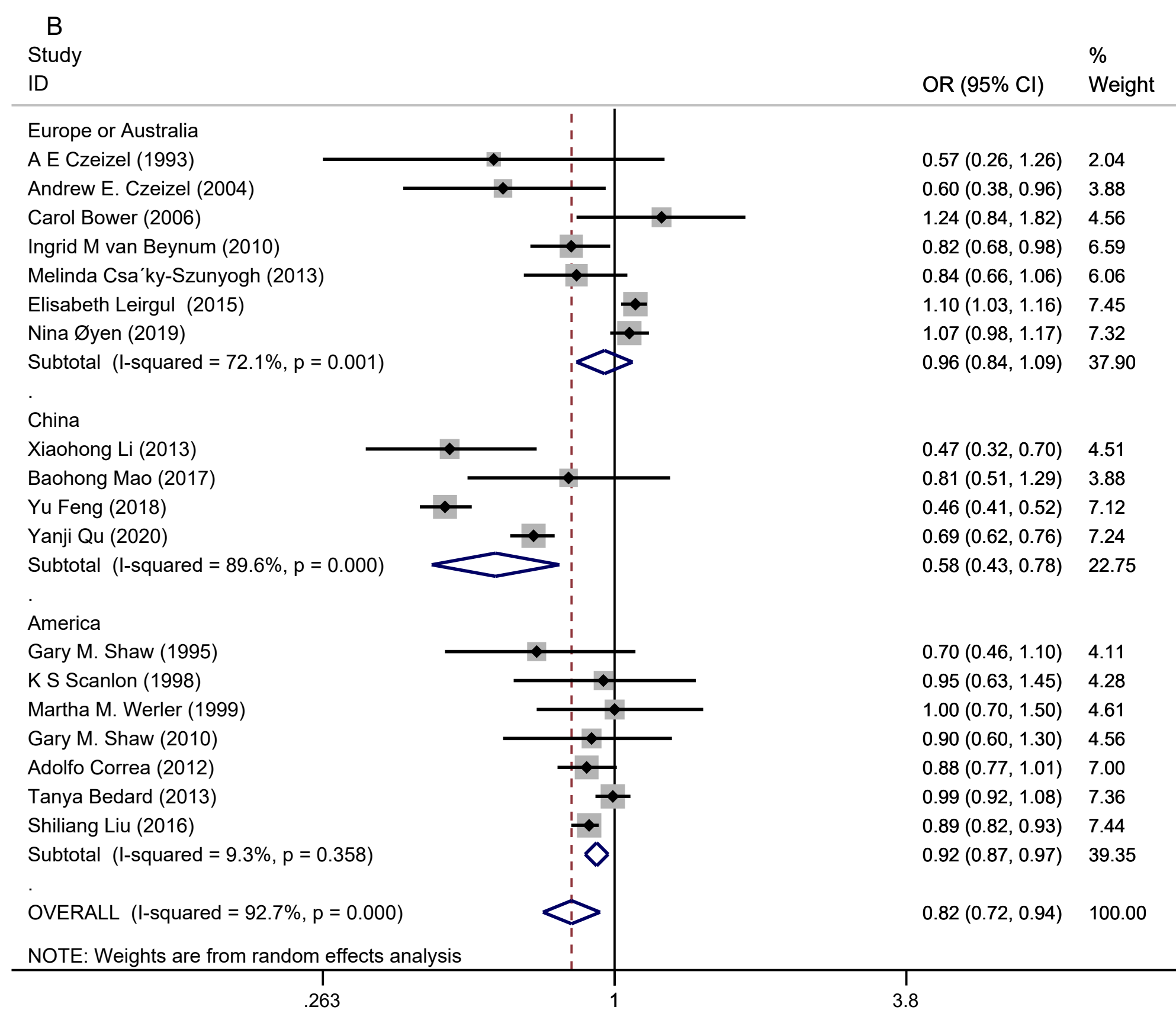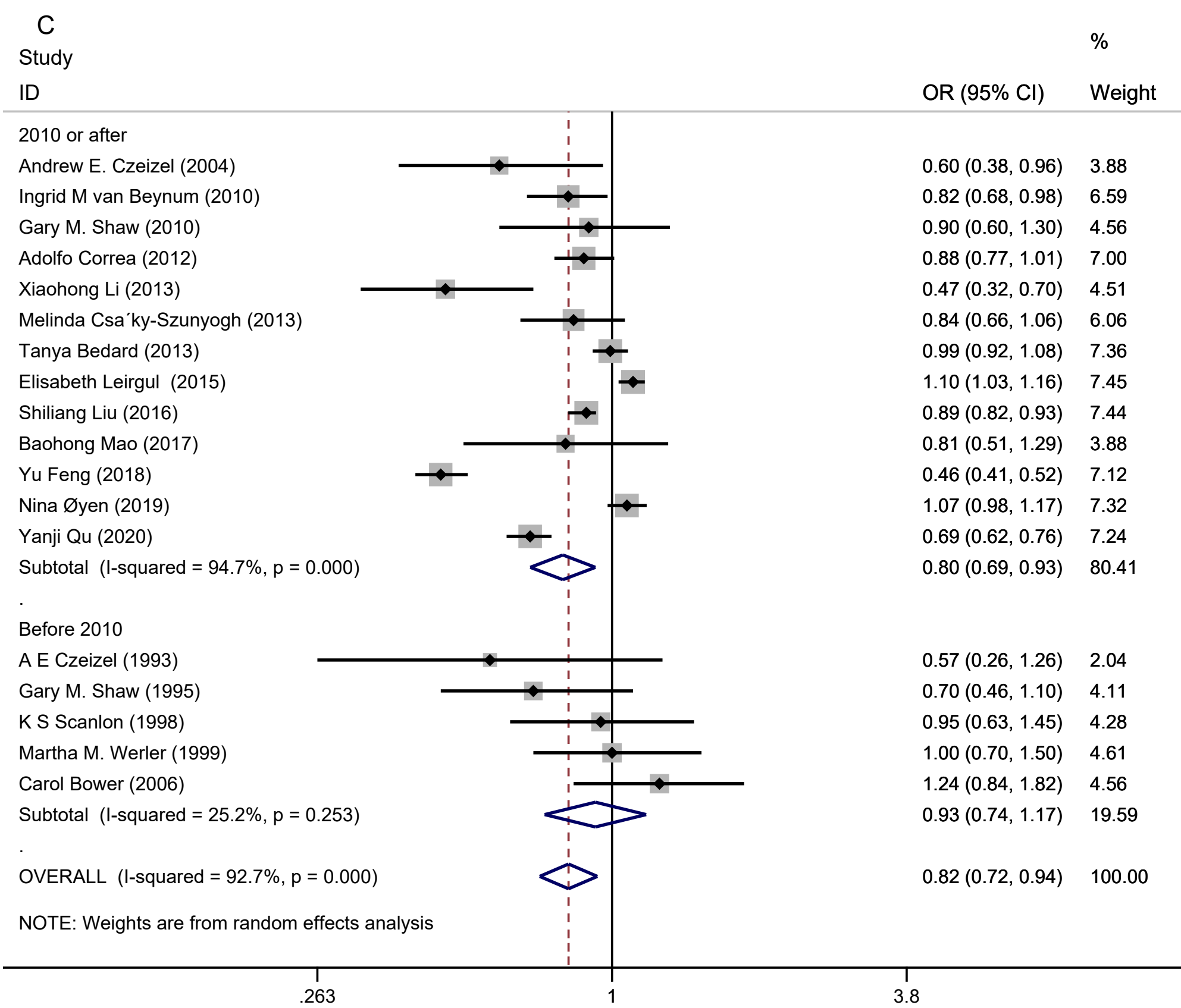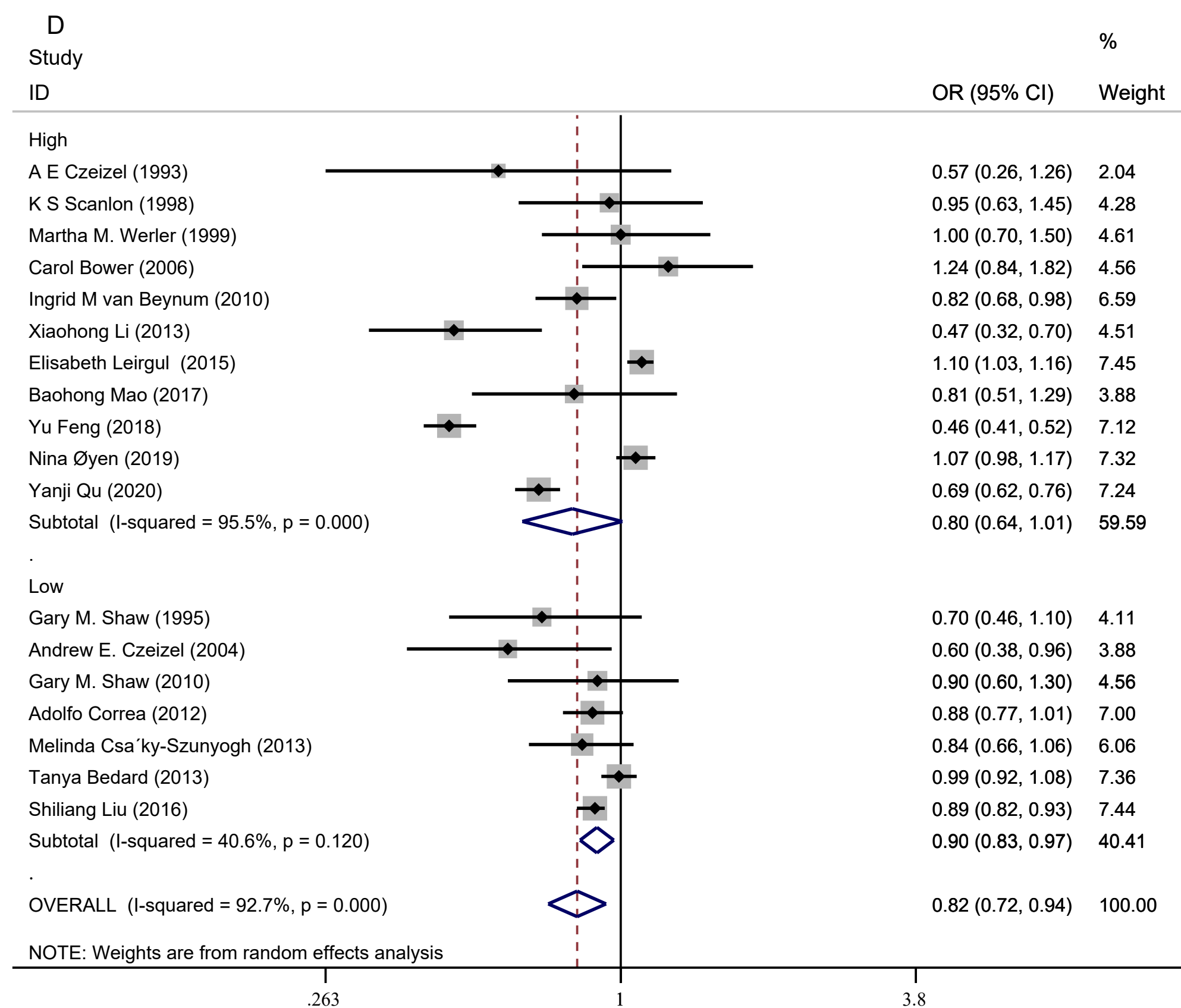

Supplement: Supplementary file 2 — Additional file 2. [file 12937_2022_772_MOESM2_ESM.pdf]

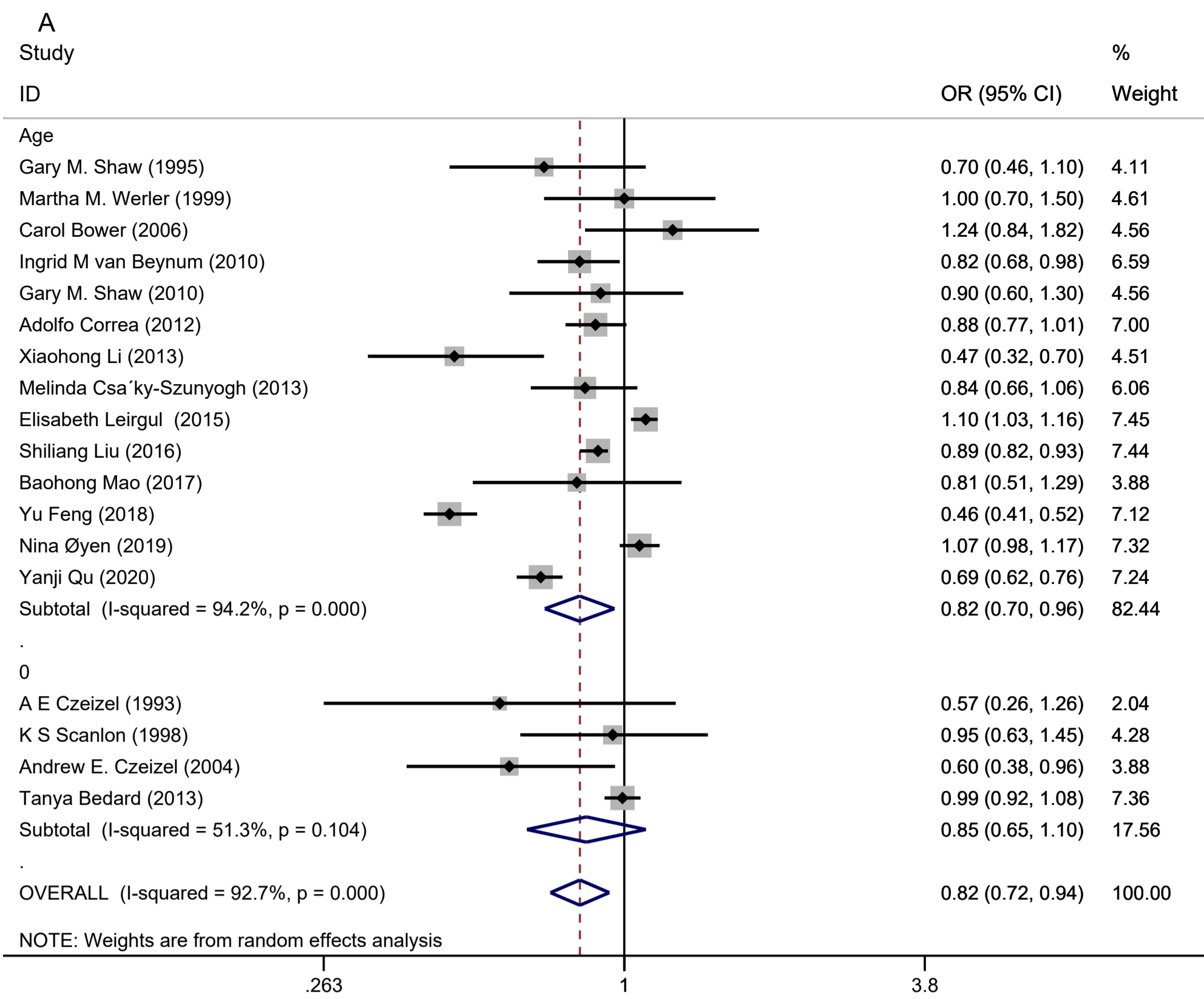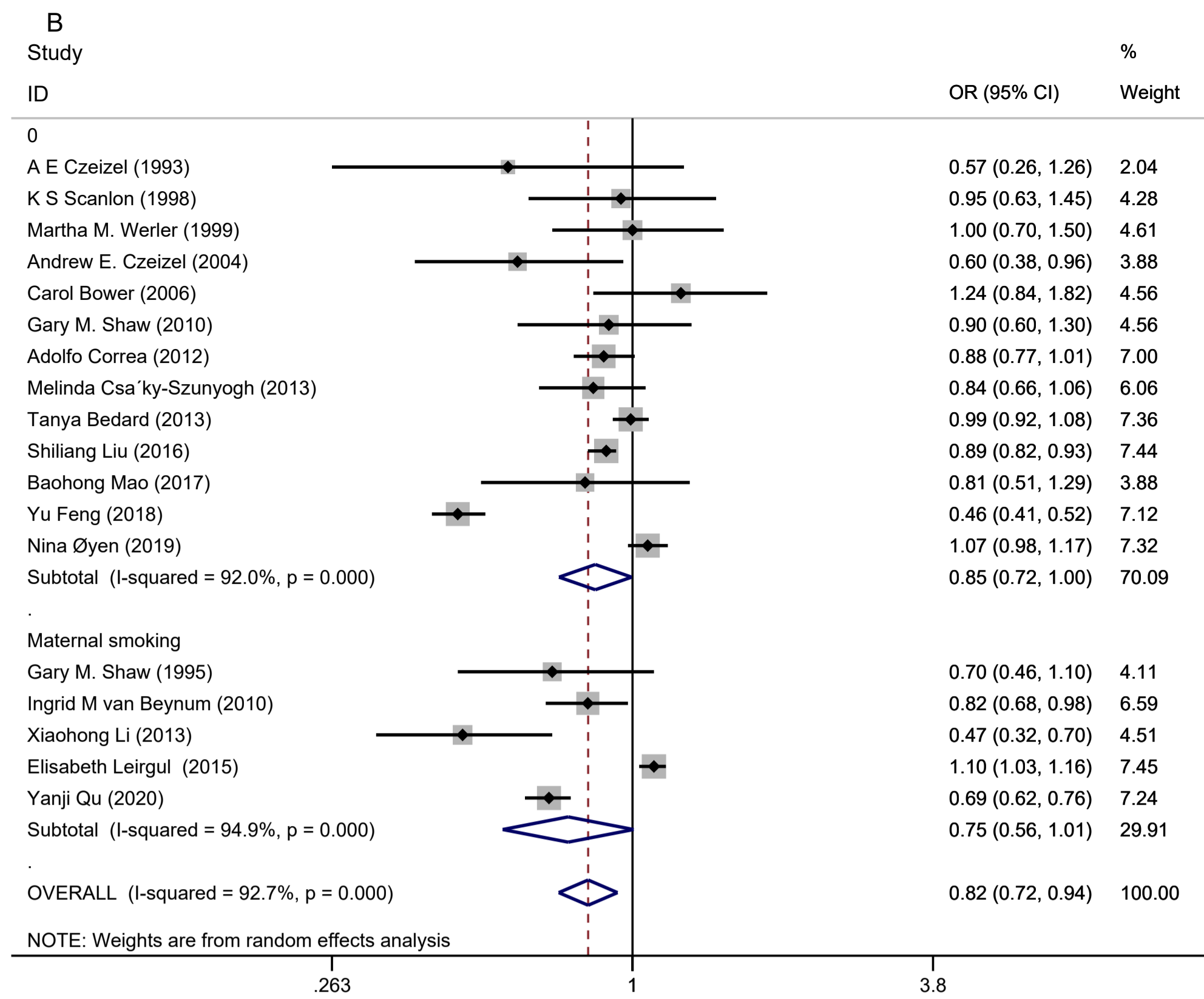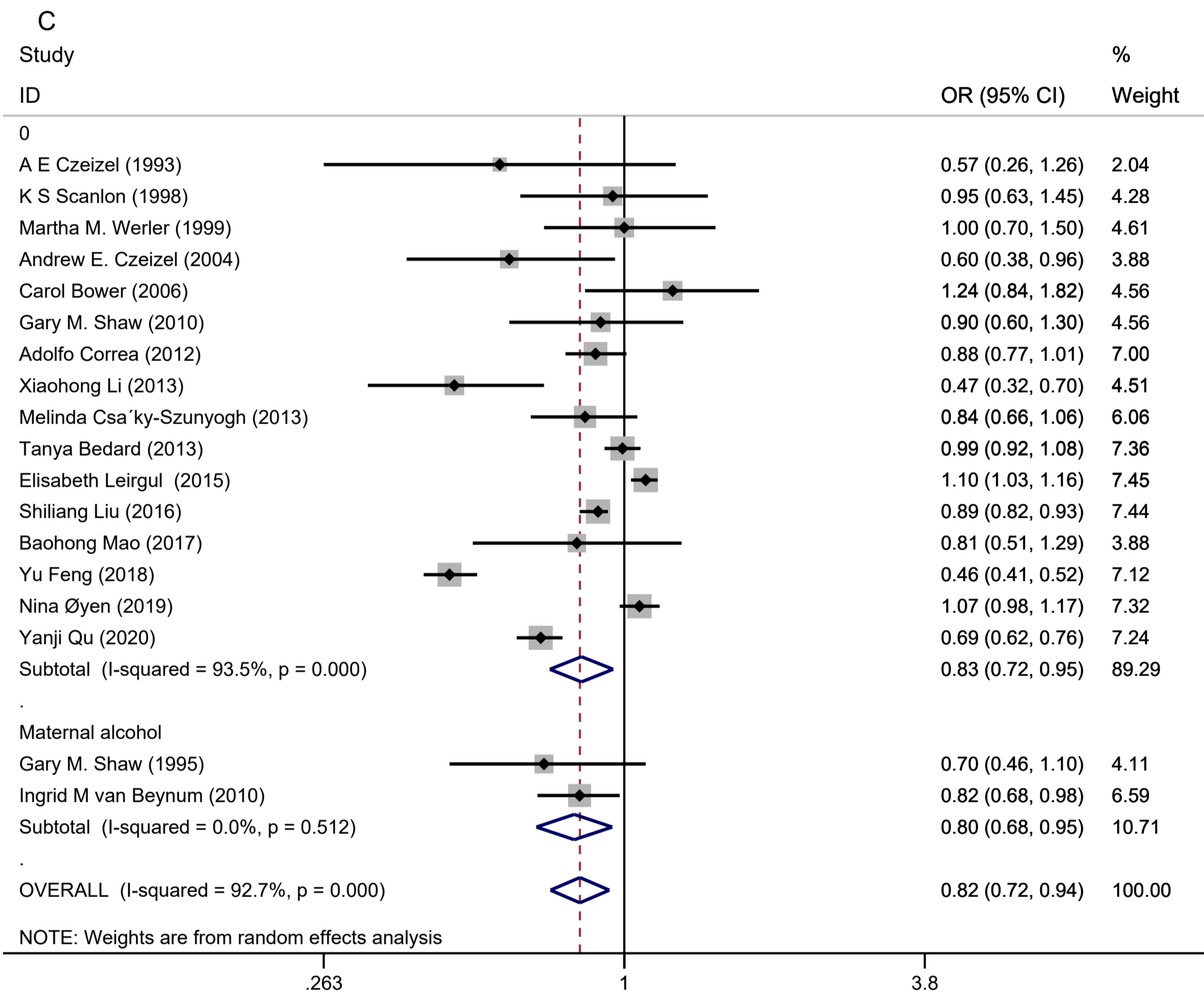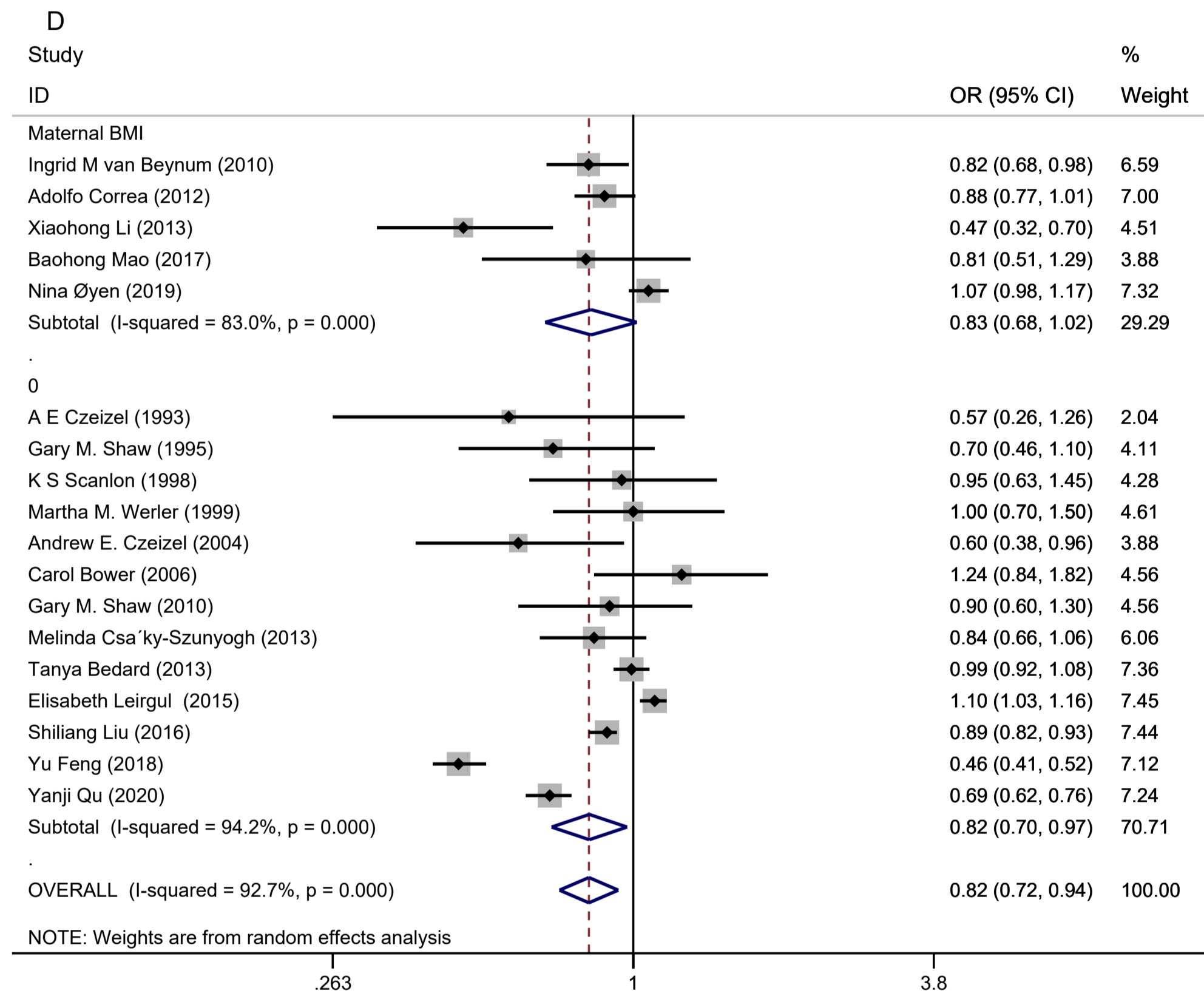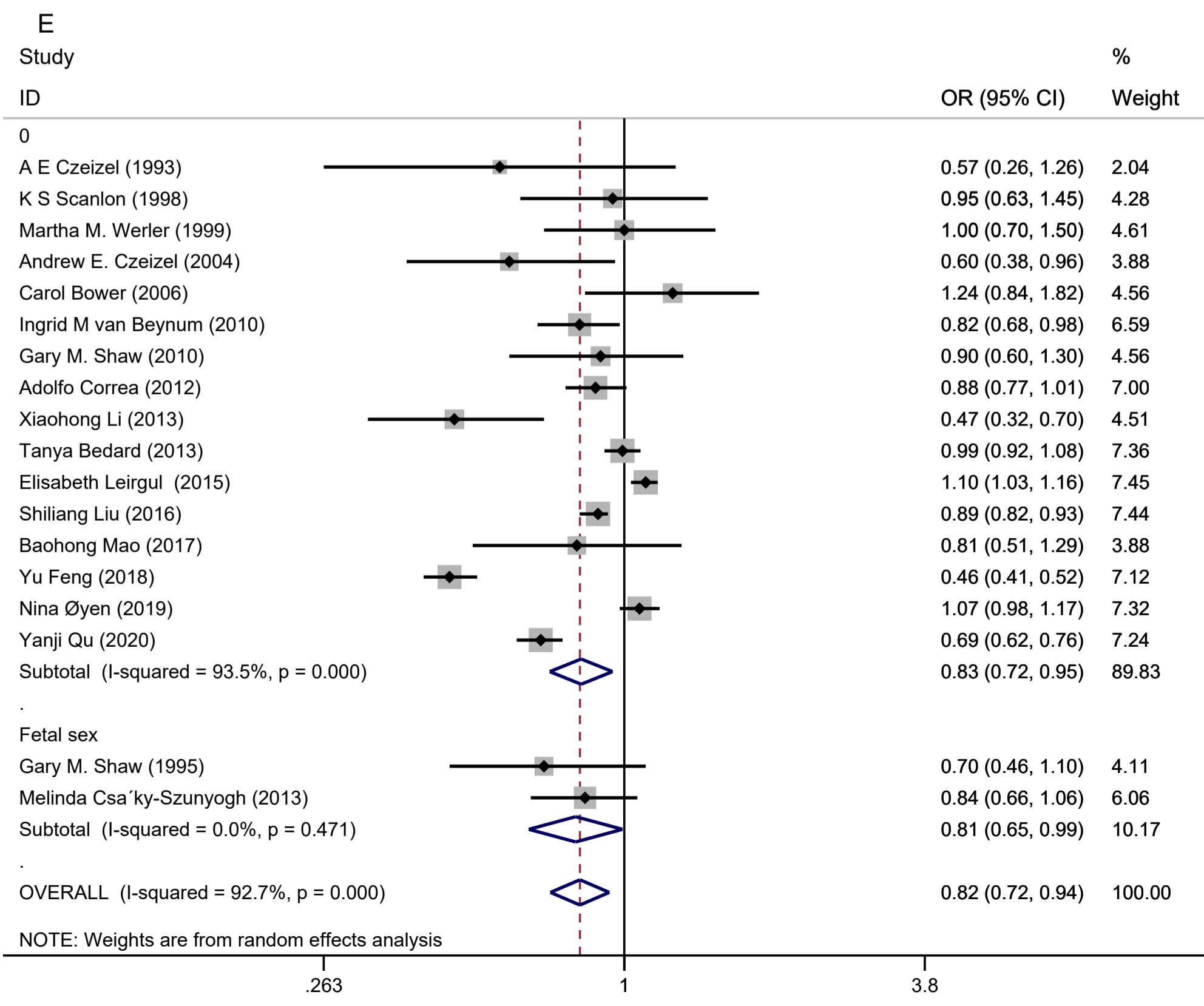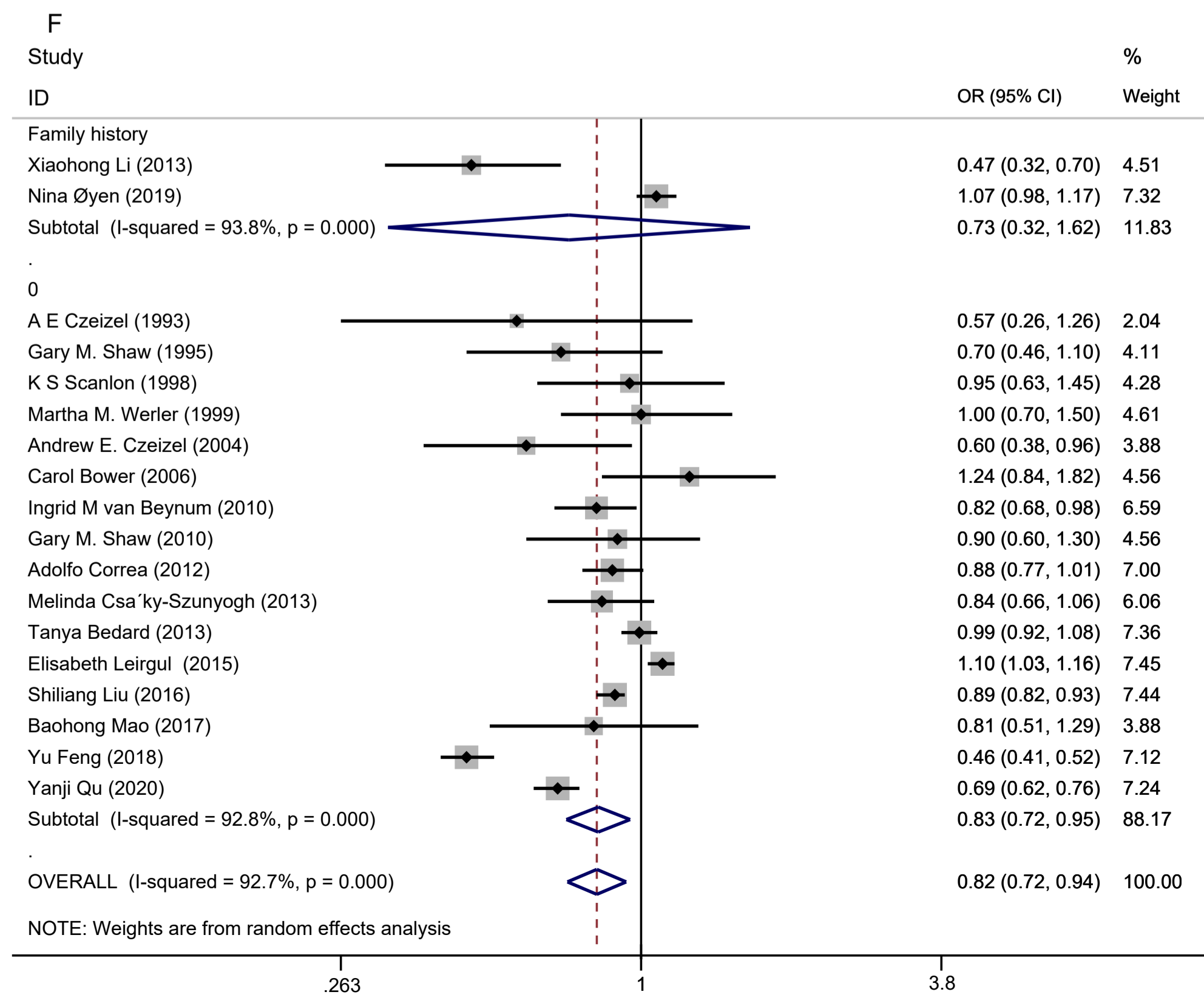

Supplement: Supplementary file 3 — Additional file 3. [file 12937_2022_772_MOESM3_ESM.pdf]

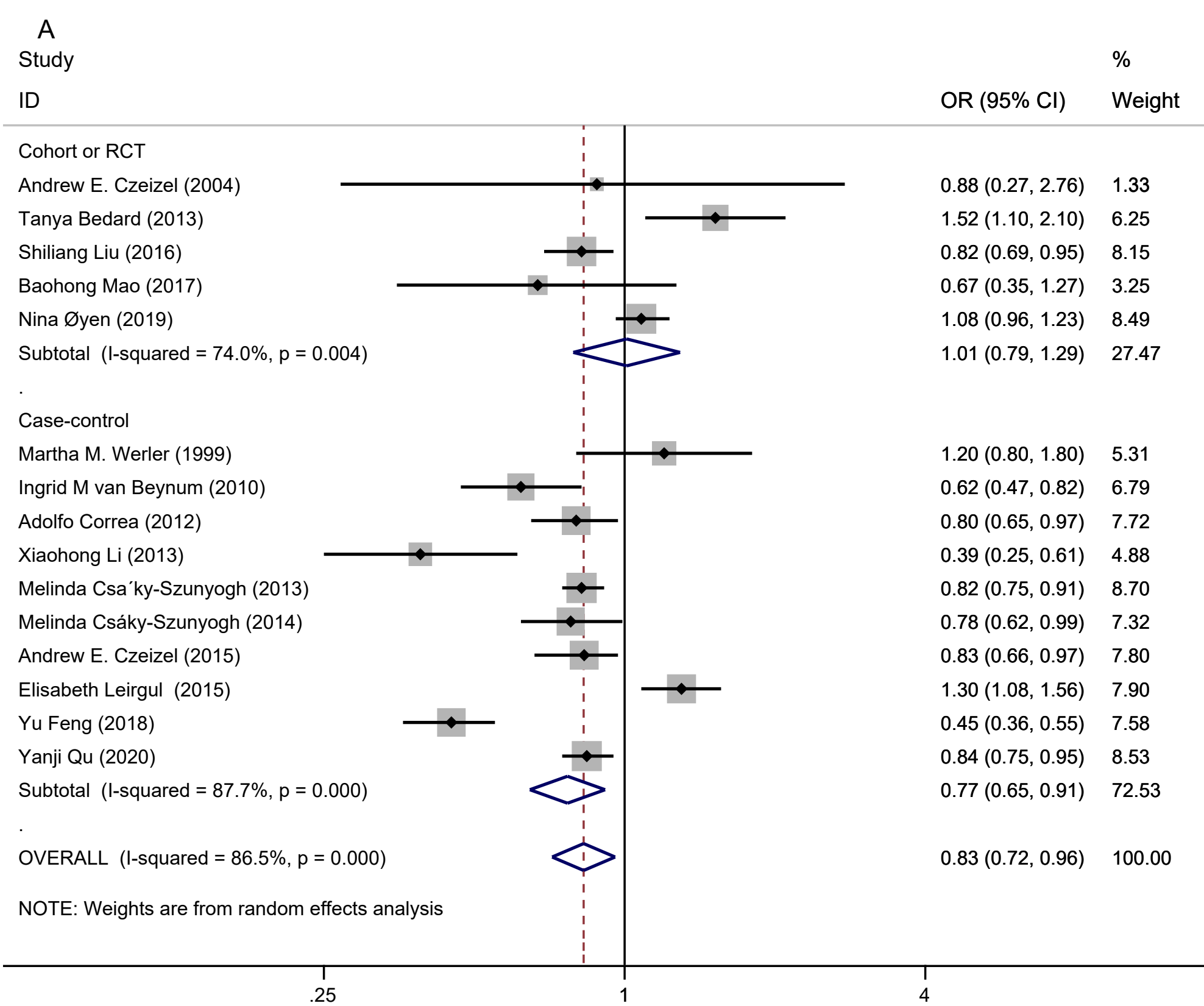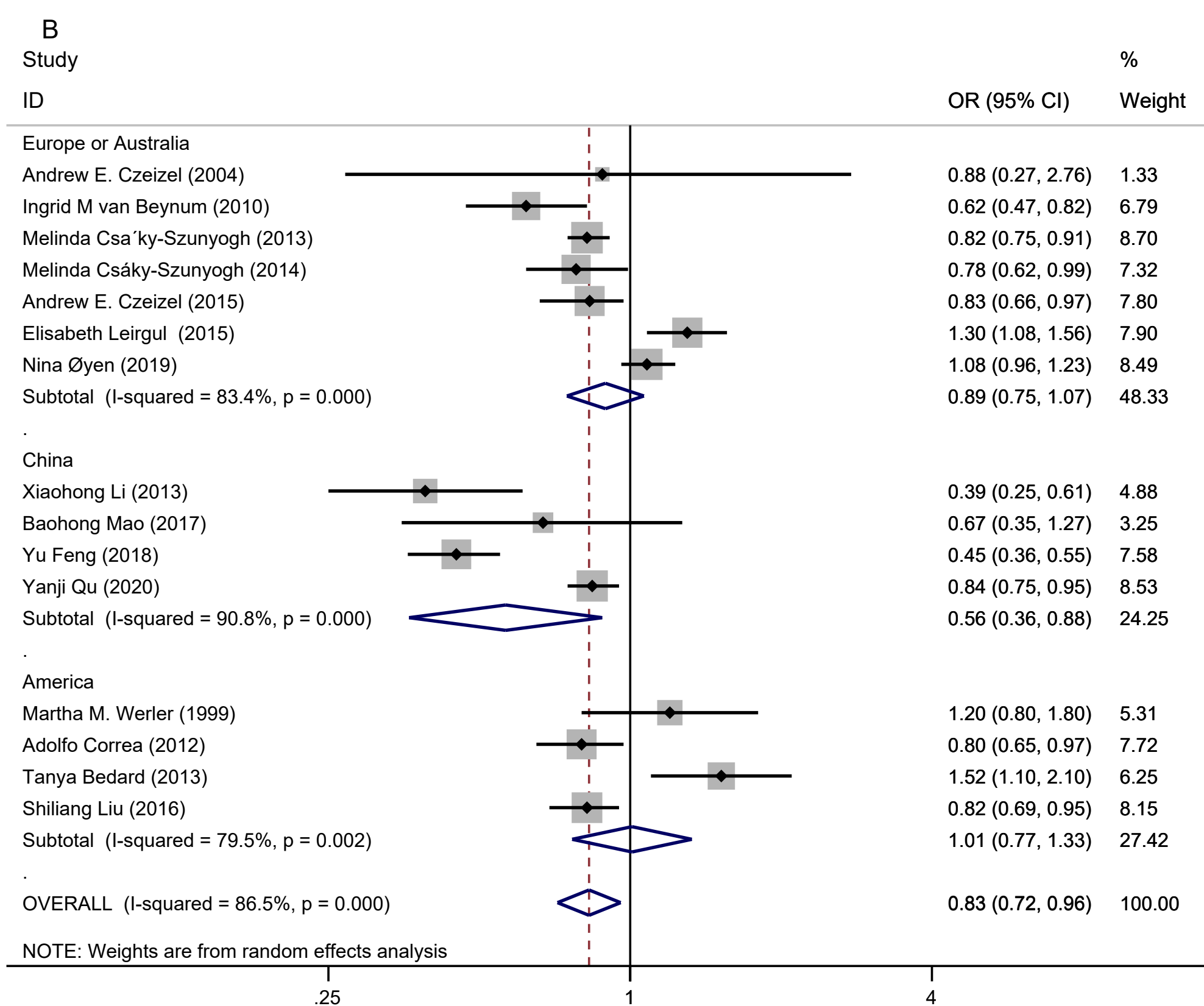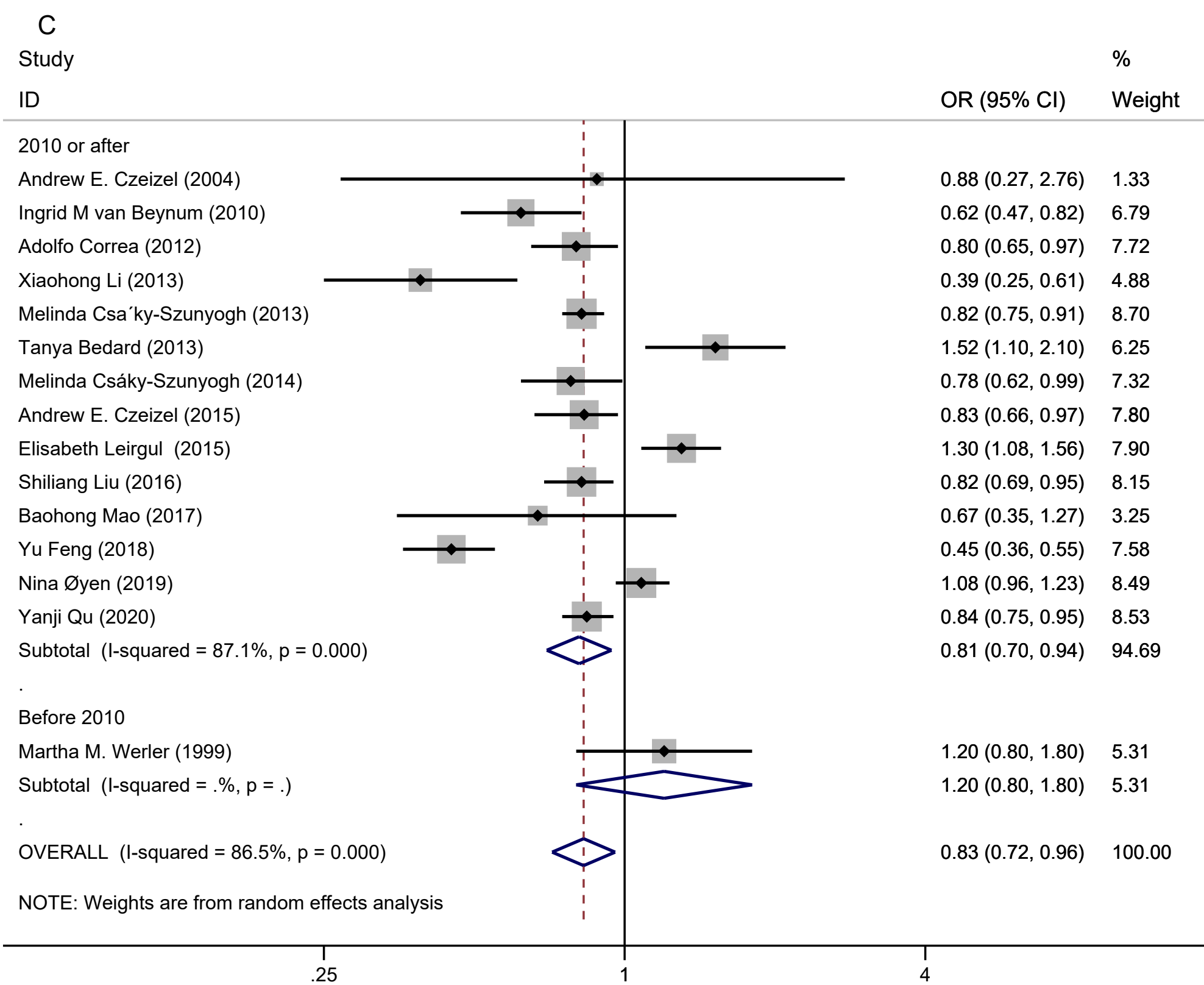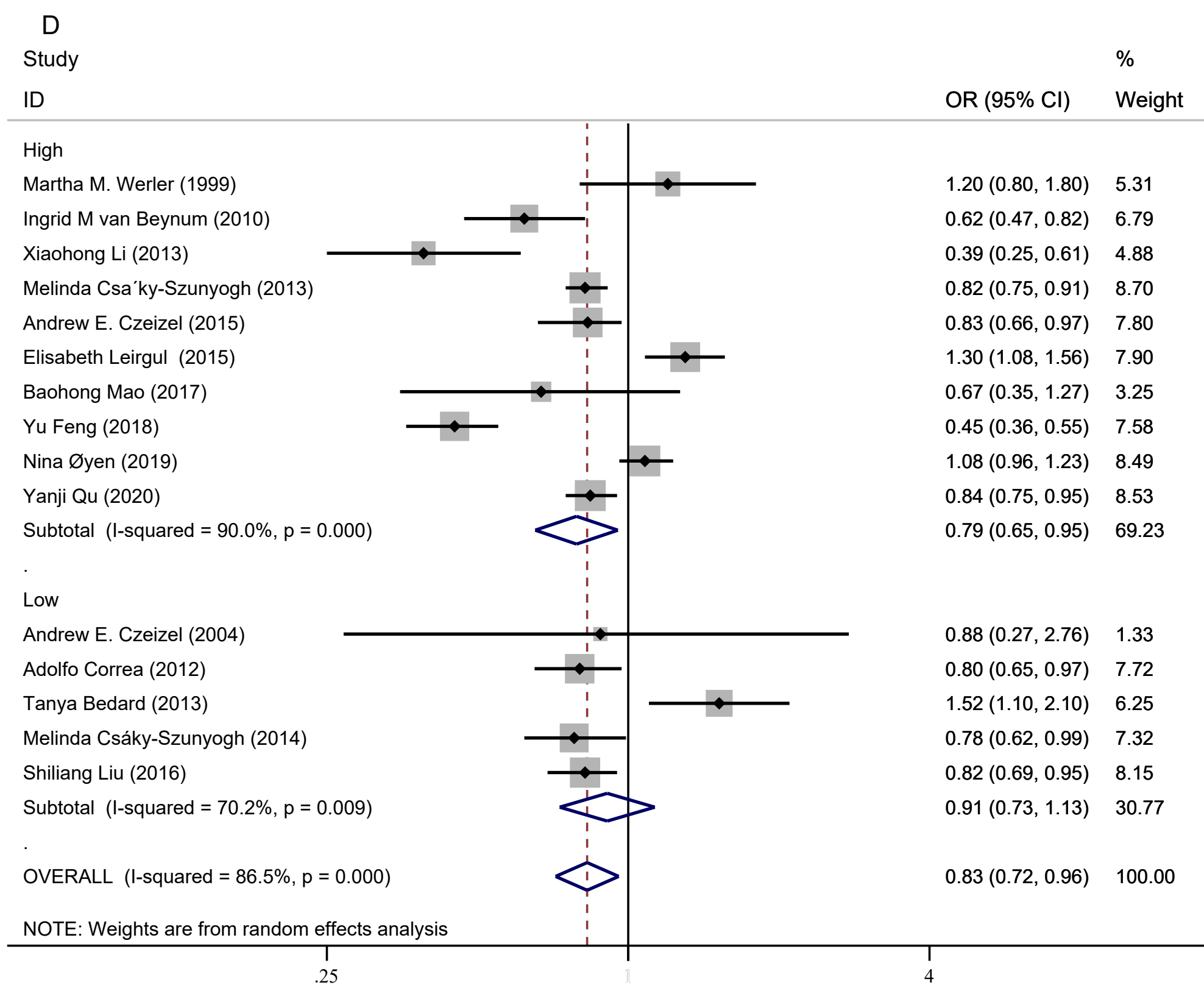

Supplement: Supplementary file 4 — Additional file 4. [file 12937_2022_772_MOESM4_ESM.pdf]

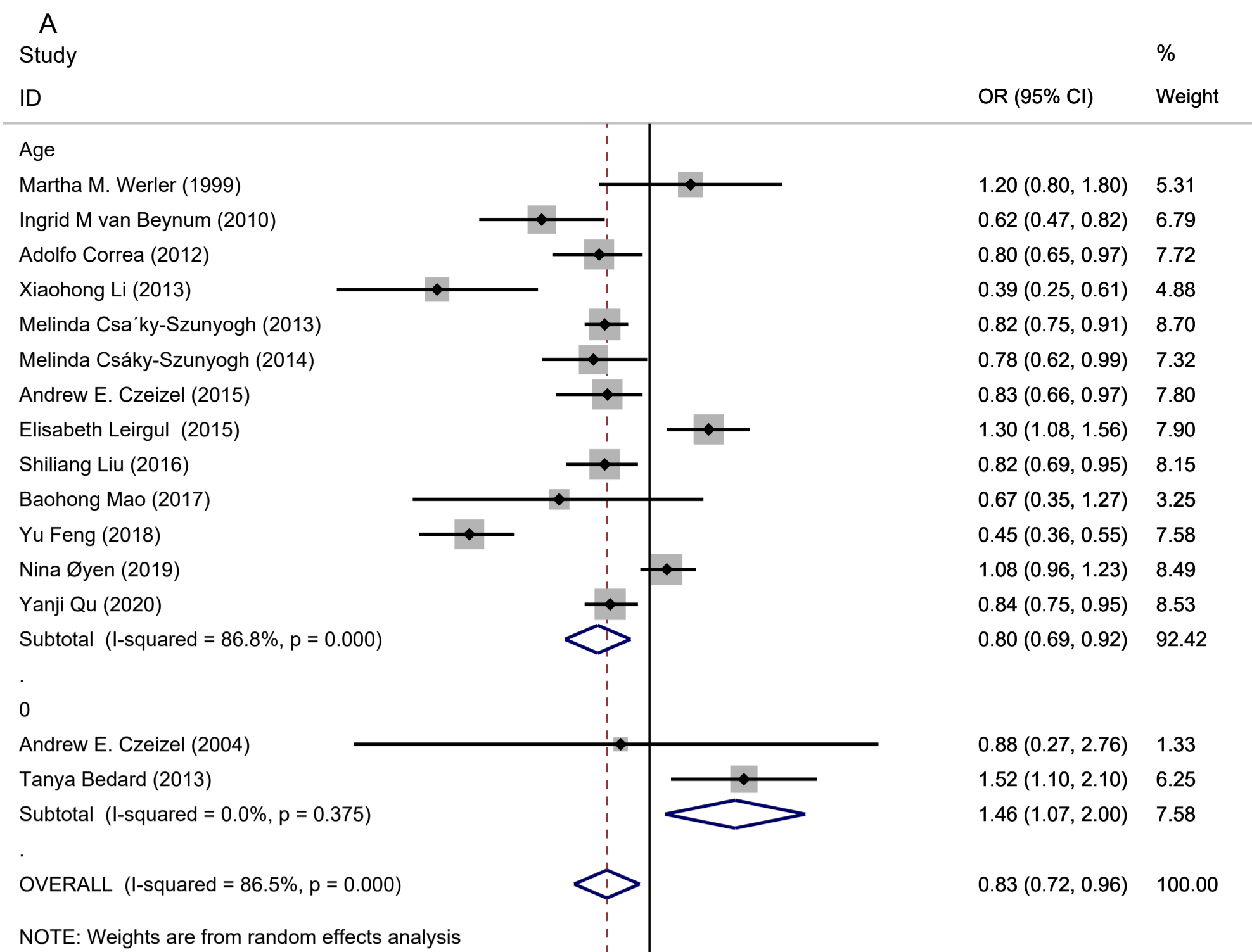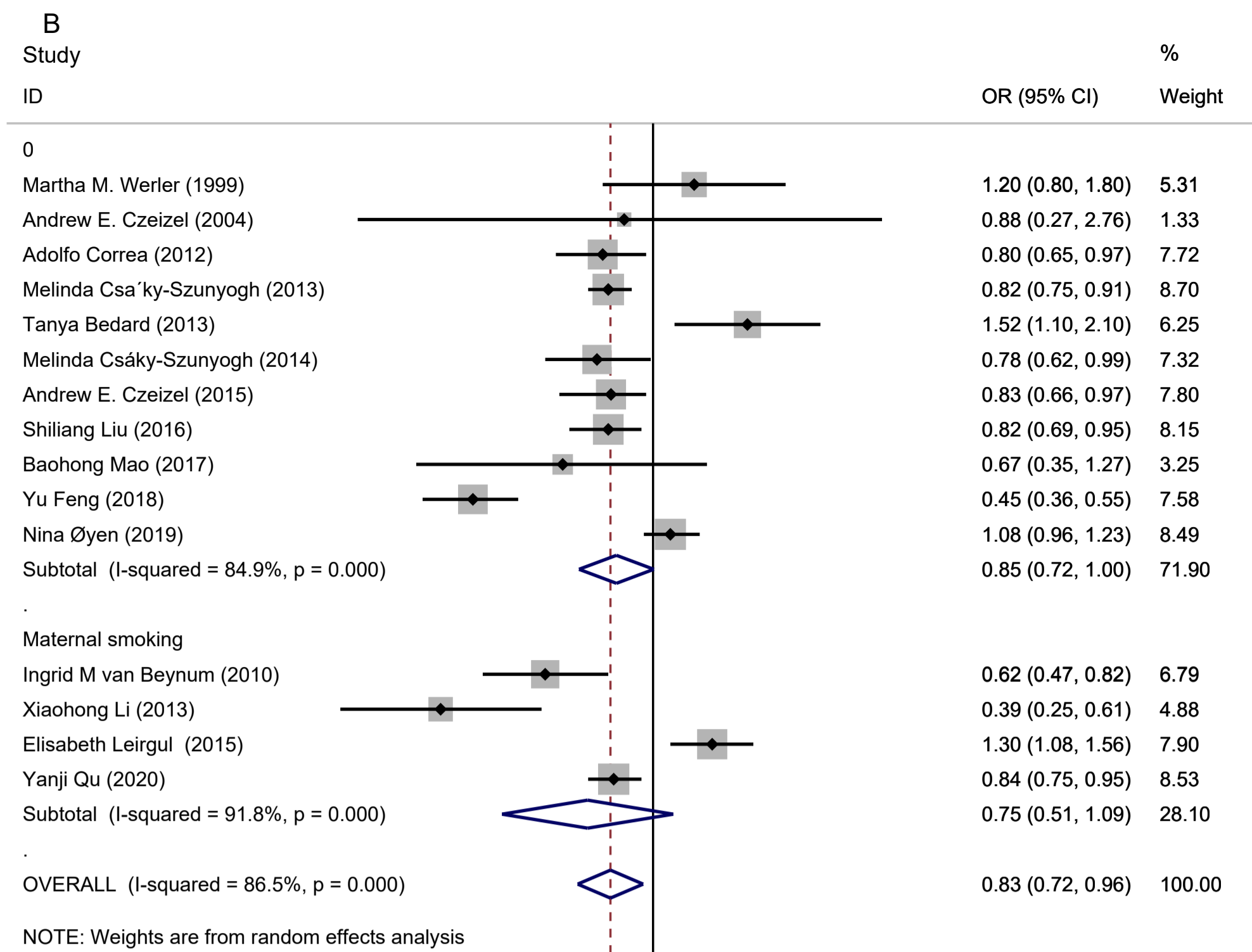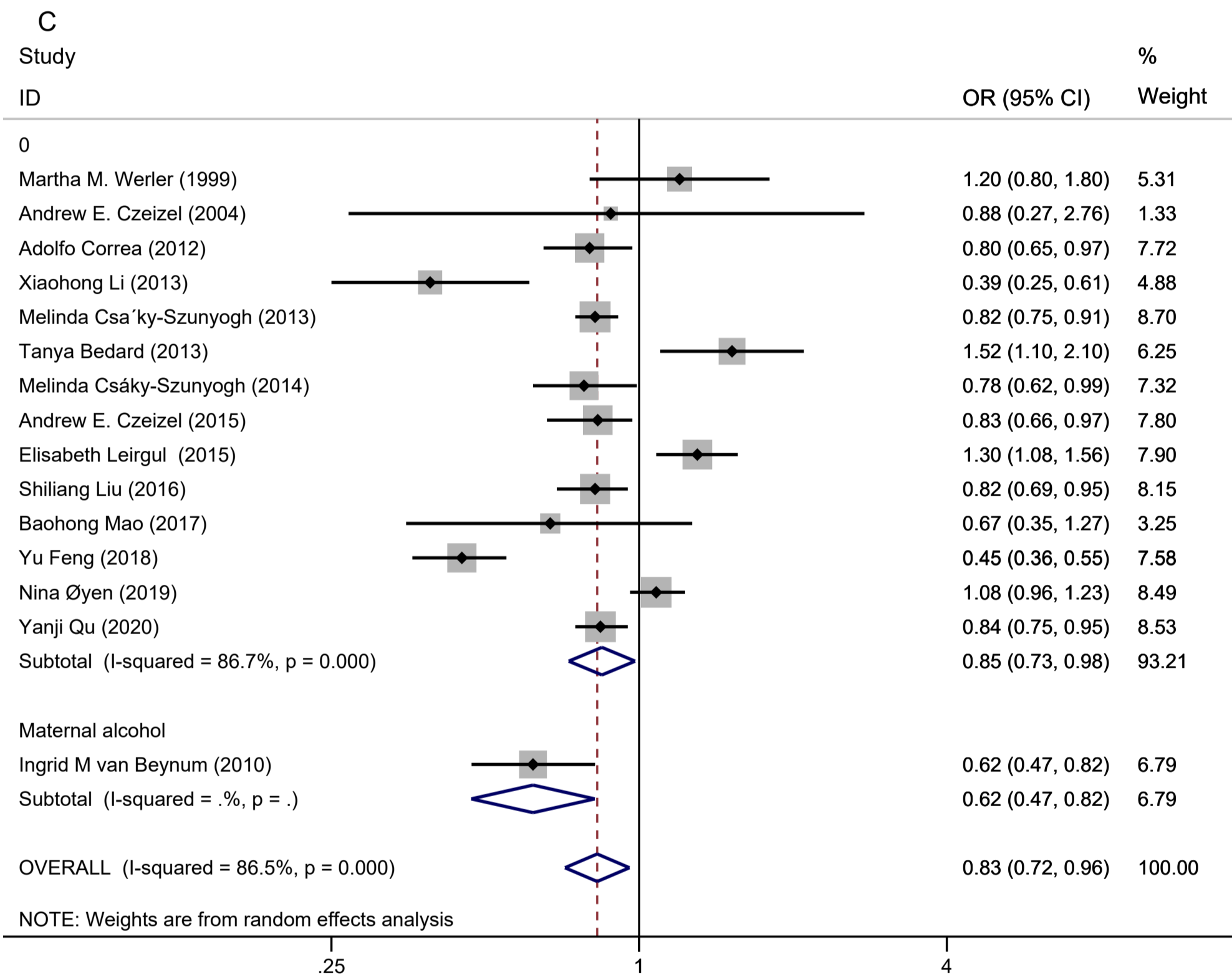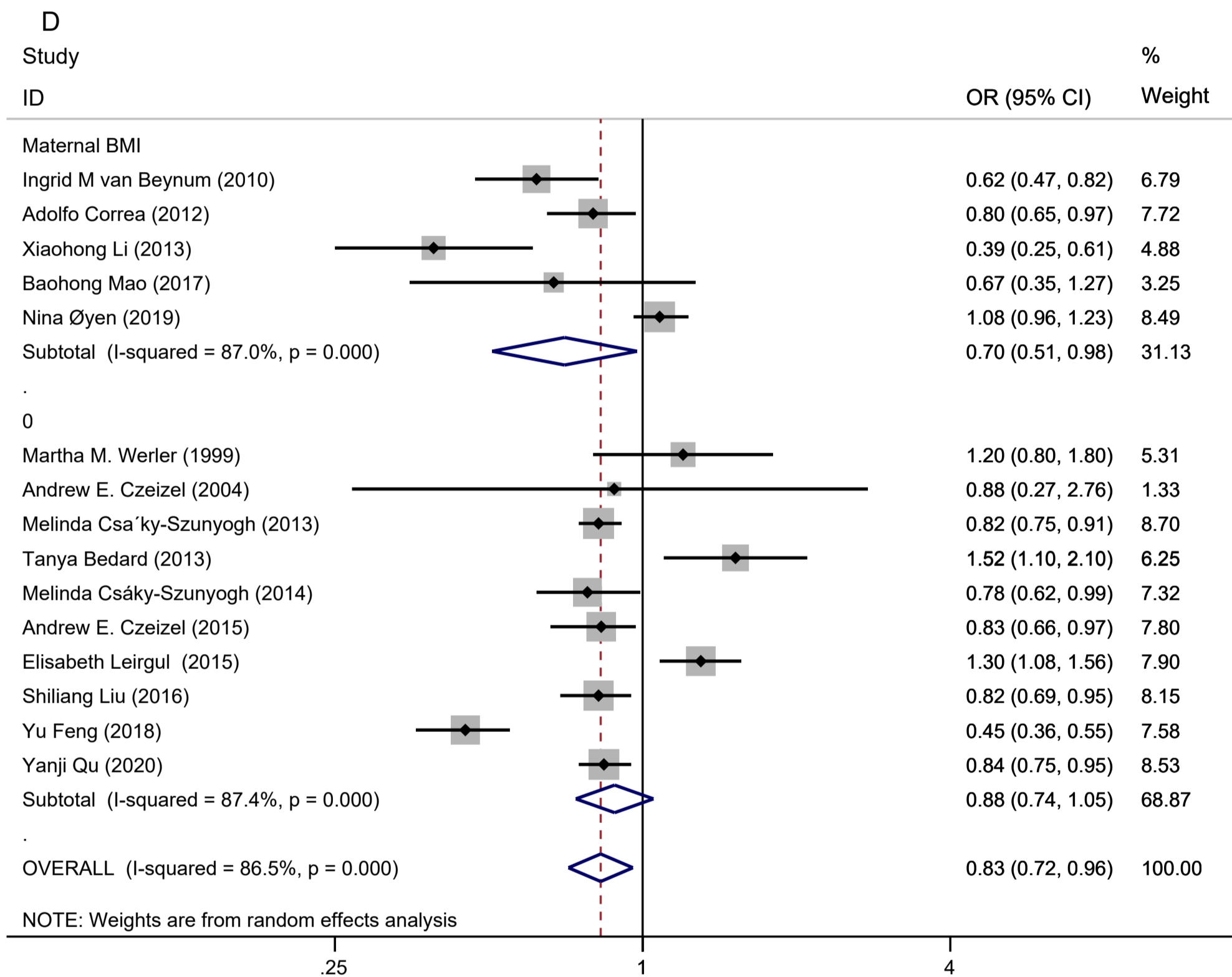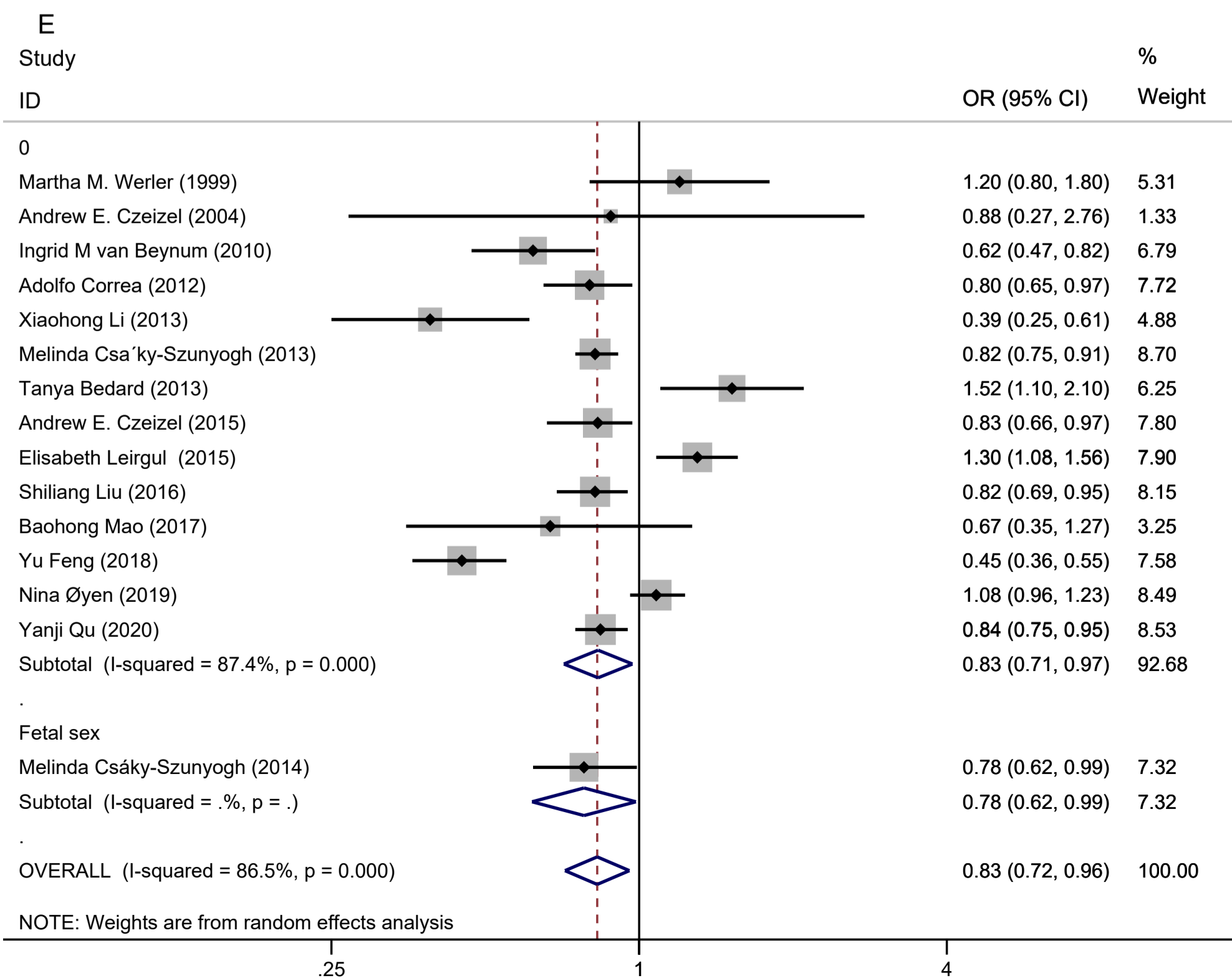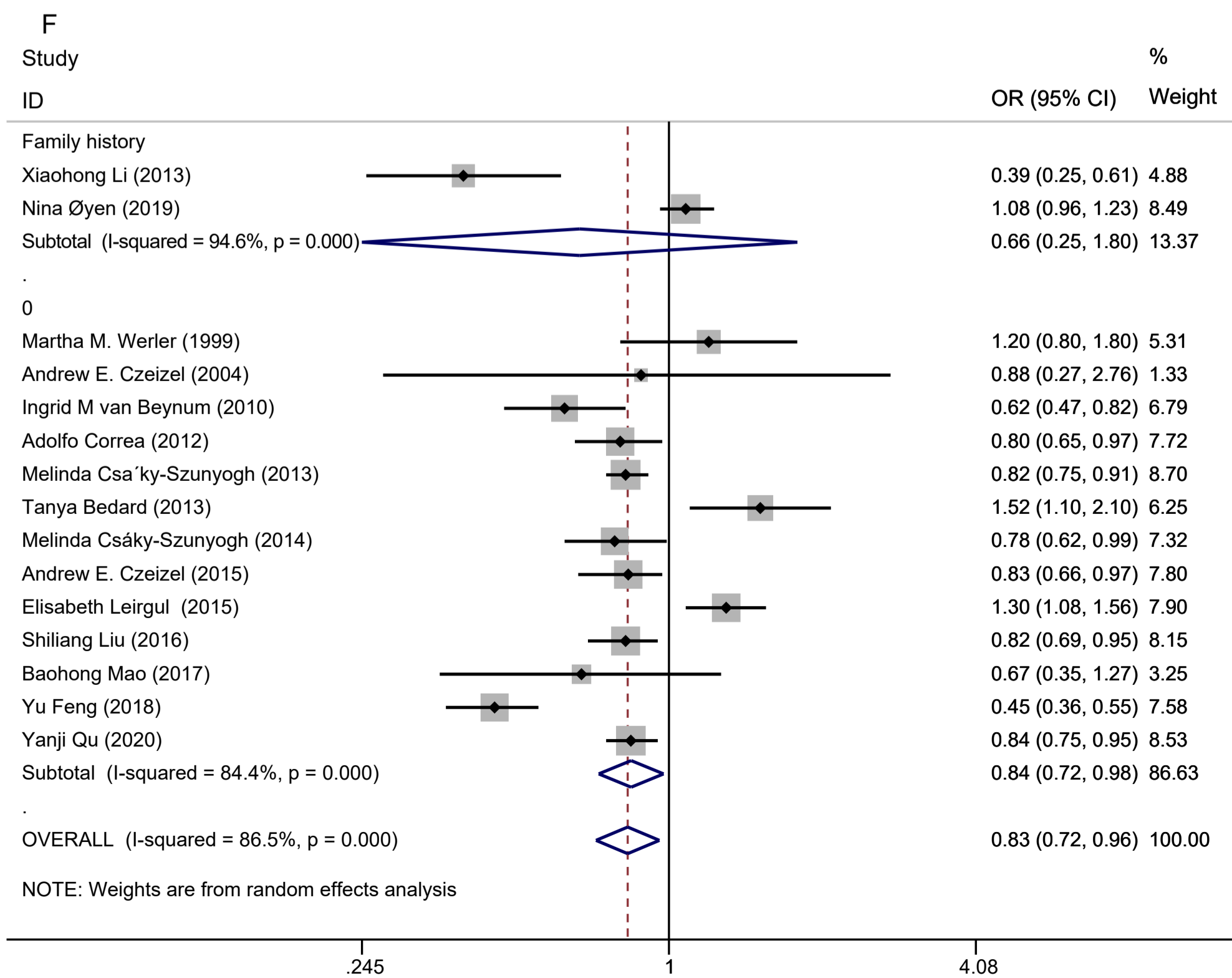

Supplement: Supplementary file 5 — Additional file 5. [file 12937_2022_772_MOESM5_ESM.pdf]
